# Adding the third dimension to studies of parallel evolution of morphology and function: An exploration based on parapatric lake‐stream stickleback

**DOI:** 10.1002/ece3.6929

**Published:** 2020-11-17

**Authors:** Grant E. Haines, Yoel E. Stuart, Dieta Hanson, Tania Tasneem, Daniel I. Bolnick, Hans C. E. Larsson, Andrew P. Hendry

**Affiliations:** ^1^ Redpath Museum and Department of Biology McGill University Montréal QC Canada; ^2^ Department of Biology Loyola University Chicago IL USA; ^3^ Kealing Middle School Austin Independent School District Austin TX USA; ^4^ Department of Ecology and Evolutionary Biology University of Connecticut Storrs CT USA

## Abstract

Recent methodological advances have led to a rapid expansion of evolutionary studies employing three‐dimensional landmark‐based geometric morphometrics (GM). GM methods generally enable researchers to capture and compare complex shape phenotypes, and to quantify their relationship to environmental gradients. However, some recent studies have shown that the common, inexpensive, and relatively rapid two‐dimensional GM methods can distort important information and produce misleading results because they cannot capture variation in the depth (*Z*) dimension. We use micro‐CT scanned threespine stickleback (*Gasterosteus aculeatus* Linnaeus, 1758) from six parapatric lake‐stream populations on Vancouver Island, British Columbia, to test whether the loss of the depth dimension in 2D GM studies results in misleading interpretations of parallel evolution. Using joint locations described with 2D or 3D landmarks, we compare results from separate 2D and 3D shape spaces, from a combined 2D‐3D shape space, and from estimates of biomechanical function. We show that, although shape is distorted enough in 2D projections to strongly influence the interpretation of morphological parallelism, estimates of biomechanical function are relatively robust to the loss of the Z dimension.

## Graphical abstract

The use of methods to acquire 3D morphological data is becoming increasingly widespread, but remains expensive and time consuming. We investigated whether 2D morphometric landmark data, which can be acquired much more quickly and with less expensive equipment, distorts interpretation of parallel evolution in threespine stickleback. Separately, we test whether morphological parallelism is distorted by 2D data, and whether calculations of biomechanical function derived from 2D data are affected by the loss of the width dimension.

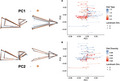

## 1 INTRODUCTION

In evolutionary biology, geometric morphometric (GM) studies of shape were an important innovation because they explicitly acknowledged the positional and functional nonindependence of traits (Caldecutt & Adams, 1998; Walker, 1997; Zelditch et al., 2012a). The vast majority of studies that have since used these GM methods have relied on two‐dimensional measurements of landmark positions. However, morphologies are not typically planar, and therefore, information will be lost using 2D measurements. Although it is true by definition that ignoring the 3‐dimensionality of biological structures results in the loss of morphological information (Roth, 1993), it is not clear to what extent ignoring this third dimension could influence interpretations of evolutionary patterns within species. Here, we consider how two‐dimensional (2D) versus three‐dimensional (3D) landmark representations might alter inferences regarding parallelism of evolutionary changes in morphology and function.

With respect to morphology, a few studies have compared 2D versus 3D GM techniques, showing that 2D projections distort shape differences—in some cases rather severely (Álvarez & Perez, 2013; Buser et al., 2018; Cardini, 2014; McWhinnie & Parsons, 2019; Santana et al., 2019). Buser et al. (2018), for instance, showed that incorporating head width as a third dimension altered the outcome of tests relating morphology in sculpins to prey evasiveness. Further, in threespine stickleback (*Gasterosteus aculeatus* Linnaeus, 1758), 3D GM methods have proven superior to 2D GM methods in identifying quantitative trait loci associated with the phenotypic differences between anadromous and freshwater populations (Jamniczky et al., 2015). Broadly, these and other differences between 2D and 3D morphological results demonstrate the consequences of ignoring variation in the dimension perpendicular to 2D projections of shape (i.e., the Z dimension), which—at least in fish—is usually the width of the animal. Yet this Z dimension is expected to be critical for some inferences, given that studies employing univariate morphometric measurements or indices, such as suction index and epaxial width, have shown important evolutionary differences in trophic function related specifically to width (McGee et al., 2013, e.g., gape width, Reimchen & Nosil, 2006).

Moving from morphology to function (the way in which an organism interacts with its environment) projecting 3D structures into two dimensions imposes artificial constraints on predicted motion—constraints that are less severe in the original 3D space. This loss of information can cause problems when estimating function because it distorts the size, orientation, and spatial relationships of the various components of a given structure. As a concrete example, the opercular and maxillary four‐bar linkages in teleost fishes are used to study the consequences of many‐to‐one form‐to‐function mapping (Alfaro et al., 2004, 2005; Thompson et al., 2017), in which multiple phenotypes can produce similar functions (Wainwright et al., 2005, but see Cooper & Westneat, 2009). Simple calculations of the mechanical efficiency of these four‐bar lever linkages require the assumption that all bars exist in a single plane and all joints are restricted to rotation within that plane (Alfaro et al., 2004; Anker, 1974; Westneat, 1990). Yet the movement of these joints is decidedly not coplanar, and the disparity between the planar simplifications of these four‐bar linkages and the 3D reality can dramatically alter expected kinematic outcomes (Olsen et al., 2017; Olsen & Westneat, 2016).

Given these possible problems in morphometric or functional analyses resulting from the collection of 2D morphological data from 3D structures, important evolutionary insights could be compromised in 2D studies. Our goal in the present paper is to estimate just how large this problem might be through its evaluation in the context of a classic evolutionary question—parallel evolution, also known in related guises as convergent evolution, predictability, or repeatability (Conte et al., 2012; Lenski & Travisano, 1994; Losos, 2010). Study systems in which populations evolve in similar ways in response to similar environments (i.e., in parallel) are important indicators that selection has some crucial deterministic effects on evolution despite the potential complicating influence of other processes or circumstances, such as gene flow, drift, and genetic bottlenecks (Hendry & Taylor, 2004; Keller & Taylor, 2008). Hence, we explicitly evaluate the extent to which the usually missing third dimension is critical to interpretations in a system frequently used to assess parallel evolution.

### 1.1 Study system

Studies of parallel evolution in threespine stickleback have often focused on trophic morphology, which exhibits heritable variation that evolves in response to prey type, and thus varies between habitat types with different prey communities (Hart & Gill, 1994; Oke et al., 2016; Schluter & McPhail, 1992). For instance, many studies have examined body shape and head shape in ecotype pairs, such as adjacent lake‐stream (Berner et al., 2008; Deagle et al., 2012; Ravinet et al., 2013), benthic‐limnetic (Gow et al., 2008; McPhail, 1992), or mud‐lava (Kristjánsson et al., 2002) populations. The parapatric lake‐stream populations on Vancouver Island, British Columbia, have been particularly well‐studied because they provide exceptionally high numbers of independently evolved replicates of stickleback populations across this environmental contrast (Berner et al., 2008, Berner et al., 2009, Hanson et al., 2016, Hendry & Taylor, 2004, Kaeuffer et al., 2012, Lavin & McPhail, 1993, Stuart et al., 2017, Thompson et al., 2017, Thompson et al., 1997, citations in table S1 of Weber et al., 2016). However, almost all of this work to date has been based on univariate or 2D GM methods, including all previous estimations of functional divergence. While adaptation of trophic function is described using a variety of metrics, including suction index, epaxial width, jaw protrusion, and kinematic transmission of lever linkages, which generally relate to feeding performance on different prey items (Jamniczky et al., 2014; Schmid et al., 2019; Thompson et al., 2017), it is not clear how well these metrics derived from 2D measurements represent the interaction between a stickleback and its prey that occurs in 3D space.

We use this lake‐stream stickleback system to analyze how inferences about parallel evolution—of both morphology and function—are influenced by explicit recognition and incorporation of the third (*Z* = width) dimension of morphology. Using multiple lake and stream fish from each of six lake‐stream population pairs, we generate new 2D and 3D GM datasets obtained from the same micro‐computed tomography (µCT) scans to ask three questions. (1) Does loss of the Z dimension when using 2D morphometrics result in substantial loss of information when compared with 3D geometric morphometrics? (2) Does loss of the Z dimension alter interpretations regarding the parallel evolution of shape? (3) Does use of 2D data to estimate biomechanical functions distort patterns of parallel functional evolution? Because parallel adaptive responses occur along continuous environmental gradients—and in the present case along gradients of available prey—we used diet data to calculate the position of each population along prey type (“stream‐like” vs. “lake‐like”) and diversity (“generalist” vs. “specialist”) axes. These axes were used in analyses of parallelism in addition to the traditional “lake” and “stream” habitat factors.

## 2 MATERIALS AND METHODS

### 2.1 Specimens, µCT Scanning, and 3D Mesh Preparation

Threespine stickleback specimens were collected by minnow trapping from May‐July 2013 from five watersheds (Beaver, Boot, Misty, Pye, and Roberts) on Vancouver Island, British Columbia, and a sixth watershed (Village Bay) from nearby Quadra Island. Fish were euthanized with MS‐222 and preserved in 95% ethanol. In each watershed, fish were trapped from lake and stream sites that represent parapatric population pairs. The lake‐stream pairs chosen are a subset of those that have been the subject of extensive research related to parallel evolution (Berner et al., 2008, 2009; Hanson et al., 2016; Hendry & Taylor, 2004; Kaeuffer et al., 2012; Paccard et al., 2019; Stuart et al., 2017; Thompson et al., 2017). Collections were made following an Animal Use Protocol from McGill University, and were permitted by the British Columbia Ministry of Forests, Lands, and Natural Resources for all populations, and Fisheries and Oceans Canada for the Misty populations, which are protected by the Canadian Species at Risk Act.

Five individuals from each site were chosen for scanning from each population, except for Pye Lake, from which only four fish were available. The sex of fish had been determined previously by Hanson et al. (2016) using spawning coloration and evidence of gravidity. When possible, a balanced sex ratio and also an immature specimen were included in the subsample for each population. Counts of specimens by watershed, habitat, and sex are shown in Table 1. We prepared specimens for µCT scanning by placing them in a standardized position, straightened against a wooden brace placed on the left side of each specimen as in Jamniczky et al. (2015). All specimens were scanned using an xRadia Versa 520 (Zeiss) at 60 V and a resolution of 20 µm. Scans were segmented and converted to 3D mesh files using Dragonfly (v. 3.6, Object Research Systems (ORS) Inc.). Using Dragonfly's mesh smoothing function, meshes were subjected to two iterations of smoothing to reduce noise and make structure boundaries more clear. We landmarked 3D meshes using the software program Meshlab (v. 2016.12, Visual Computing Lab of CNT‐ISTI; Cignoni et al., 2008). All subsequent data processing and analysis were performed in the R statistical environment (R Core Team, 2019).

**TABLE 1:** Counts of specimens by Watershed, Habitat, and Sex. The “U” column under Sex indicates specimens that were not identifiable as male or female based on morphology

Watershed	Habitat	Sex			Total
		M	F	U	
Beaver	Lake	2	2	1	5
	Stream	1	3	1	5
Boot	Lake	2	2	1	5
	Stream	2	2	1	5
Misty	Lake	2	2	1	5
	Stream	2	2	1	5
Pye	Lake	1	1	2	4
	Stream	2	2	1	5
Roberts	Lake	2	2	1	5
	Stream	2	2	1	5
Village Bay	Lake	2	1	2	5
	Stream	2	2	1	5
Total		22	23	14	59

### 2.2 Data preparation

**FIGURE 1:**
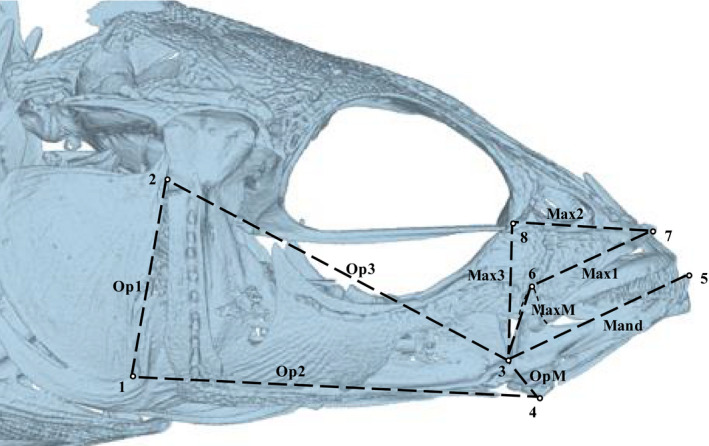
A 3D surface mesh of a µCT scanned threespine stickleback showing the lever systems that are the subjects of this study. Landmarks used for analysis are indicated by the circles, and the landmark numbers correspond to those in Table 2. The opercular 4‐bar linkage consists of lever bars Op1, Op2, Op3, and OpM. The mandibular lever system includes a closing lever (MaxM and Mand), and an opening lever (OpM and Mand). The Maxillary 4‐bar linkage consists of Max1, Max2, Max3, and MaxM. The curved dashed line of lighter weight than those used to represent the lever bars indicates the shape of the articular, which is behind several other bones

To collect landmarks to be used in analyses of 2D versus 3D morphometrics, we first placed landmarks on the surface meshes in 3D coordinate space. Landmarks were placed at the joints and ends of lever bars associated with stickleback trophic function, and each had an X (anterior‐posterior), Y (dorsal‐ventral), and Z (lateral‐medial) coordinate (Figure 1 and Table 2). Lateral landmarks were placed on both sides of each fish. For four specimens, all from different populations, some landmarks had to be interpolated using the thin plate spline (TPS) method (Adams et al., 2019) based on landmarks of other specimens within the same population. This method used all samples within a population to generate a reference set of landmarks, align the incomplete specimen's landmarks with landmarks of the reference, and then interpolate the missing landmark from its position in the reference set (Gunz et al., 2009). In most cases, landmarks were interpolated because of deterioration of scan quality near the edges of the scanner's field of view (Table S1) but, in one case, a landmark was interpolated because of a pronounced deformity of the right opercle (Figure S1).

**TABLE 2:** Landmark numbers, descriptions, and associated levers and linkages

Landmark #	Landmark description	Associated levers and linkages
1	Ventral tip of Opercle	Op4
2	Opercular‐Hyomandibular joint	Op4
3	Articular‐Quadrate joint	Op4, Mand, Max4
4	Insertion of Interopercle‐Mandibular Ligament on Angular	Op4, Mand
5	Tip of anterior‐most tooth on mandible	Mand
6	Dorsal tip of Articular	Mand, Max4
7	Anterior tip of Maxilla	Max4
8	Lateral Ethmoid‐Lacrimal joint	Max4

The landmarks placed on 3D meshes were used in both 3D and 2D GM analyses. To generate a 2D landmark dataset from the 3D landmarks, we first oriented them such that the midsagittal plane was parallel to the XY plane in coordinate space (Katz, 2017). Once specimens were rotated, Z coordinates were removed from the 3D landmarks to produce 2D coordinates of the landmarks from a lateral view of each specimen (for the rationale and details behind this approach, see Appendix S1). Once a 2D landmark dataset was generated by removing Z coordinates, the 2D and 3D landmark sets were handled separately.

We tested for asymmetry to ensure that bracing the specimens for CT scanning did not impose distortion by reflecting the landmarks on one side, Procrustes superimposing specimens, and using the *geomorph* function *bilat.symmetry*, a permutation‐based test of asymmetry (Adams et al., 2019). This test did show statistically significant directional asymmetry (consistent differences across specimens) between sides that was assumed to be a result of bracing the specimens for scanning, but it only accounted for a small proportion of the shape variation (*F*_1,58_, *R* ^2^ = .013, *p* < .001 in 2D, and *F*_1,58_, *R* ^2^ = .014, *p* = .003 in 3D). Some fluctuating asymmetry (individual differences between sides) was also present (*R* ^2^ = .067 in 2D, *R* ^2^ = .152 in 3D). Within‐specimen differences between sides were accounted for in subsequent analyses by using the mean of the bilateral landmark coordinates from both sides for each specimen.

### 2.3 Diet type and diversity

The morphological structures we used to test the effects of 2D versus 3D GM methods on parallelism are important for feeding, and consequently are presumed to adapt in parallel primarily as a response to the type of prey available (Kaeuffer et al., 2012; Schluter & McPhail, 1992; Schmid et al., 2019; Willacker et al., 2010). Although discrete habitat types are typically used to assess parallelism, the environmental characteristics that impose selection—in this case available prey types (benthic versus limnetic)—often vary in a more quantitative fashion (Berner et al., 2008, 2009; Kaeuffer et al., 2012; Stuart et al., 2017). Thus, we also used continuous variables describing diversity and types of available prey items to assess the parallelism of evolution in response to the diets of the populations we studied. Gut content data from Stuart et al. (2017) were used to characterize stickleback diets in our populations. Although these data were collected from different fish than those we scanned, they were taken from fish sampled during the same time period in the summer of 2013. Gut content data were recorded as proportions of each fish's diet made up of each food item, and common prey items included copepods, ostracods, cladocerans, chironomids, ceratopogonids, freshwater clams, caddisflies, and amphipods. See Appendix S1 Section 2 for further description of diet data.

We applied a Hellinger transformation (a square root transformation of the proportion of each prey item) before subjecting the data to a Linear Discriminant Analysis (LDA) using the R package *MASS* to discriminate gut contents between lake and stream populations (Ripley et al., 2020). The LDA described the axis of greatest divergence in diets between lake fish and stream fish, and mean values along the continuous LD axis were used to describe the diet of a population as more stream‐like or more lake‐like in subsequent analyses. We also considered the diversity of prey as a potential environmental gradient along which trophic morphology could evolve adaptations for generalist or specialist feeding. The Gini–Simpson diversity index (hereafter *Simpson*) was calculated for gut content totals within each population to describe stickleback prey diversity (Oksanen et al., 2019).

### 2.4 Trophic morphology in 2D versus 3D shape spaces

Morphological analyses were first conducted on 2D and 3D coordinate sets in separate shape spaces. Subsets of the coordinate datasets were made to represent the opercular four‐bar linkage (Op4), the maxillary four‐bar lever (Max4), and the combined opening and closing mandibular levers (Mand). Procrustes superimpositions were performed on the lever system datasets using the *gpagen* function in the *R* package *geomorph* (Adams et al., 2019). Procrustes superimposition removes information about isometric size, location, and orientation of sets of landmarks so that shapes can be compared directly. Allometric effects were controlled by performing Procrustes ANOVAs of each shape dataset against centroid size, and using the residuals for analysis. Tangent space PCAs were used to calculate the Principal component values for all plots visualizing differences between populations and between individuals in shape space.

Procrustes ANOVAs, which are tests used for shape data but are analogous to conventional linear models (Goodall, 1991), were used to test the effects of diet on shape (Adams & Collyer, 2018; Adams et al., 2019). Each of these separate tests used a diet variable as a covariate (as *habitat*, *diet LD,* or *Simpson*), and *Watershed* as a factor, and included the interaction of these terms. Additionally, because diversity and diet type were not correlated, diet diversity (as *Simpson*) was tested using *diet LD* as a covariate to test for interacting effects of diet type and diversity, in addition to additive effects. In all statistical models, Type II sums of squares were used, and main effects of diet variables (both type and diversity) were interpreted as parallel components of evolution. For more detailed discussion of these and subsequent methods, see Appendix S1 Section 3.

We calculated angles between watershed vectors (Collyer & Adams, 2018, 2019)—that is, trends through multivariate phenotype space—in morphospaces for 2D and 3D landmark datasets. In perfectly parallel adaptation between parapatric populations, the pairwise angles between all of these watershed vectors would be equal to 0°, and the angle decreases with decreasing parallelism. We also conducted pairwise comparisons of the differences in magnitudes between watersheds. Magnitude measures the units of change through morphospace per unit of a covariate. Pairwise vector angle calculations and comparisons of vector magnitudes were conducted twice, once with vectors calculated using *diet LD*, then with vectors calculated using *Simpson* as covariates.

We used two methods to quantify correlations of shape spaces between 2D and 3D landmark datasets for the three lever systems (Op4, Max4, and Mand). In the first test, we calculated the Procrustes distance of each specimen from the consensus shape. We then performed Pearson's correlation tests between each specimen's Log_10_ transformed Procrustes distance from the 2D consensus shape to its Log_10_ transformed Procrustes distance from the 3D consensus shape. In the second test, we created pairwise Procrustes distance matrices for the 2D and 3D datasets, then performed Mantel tests between them for each lever system. Mantel tests used 1,000 permutations each (Oksanen et al., 2019). In both tests, higher correlations between shape spaces imply less loss of shape fidelity after landmarks are reduced to two dimensions, but the statistical significance of these tests is not meaningful for our purposes, because we know that the 2D and 3D datasets are related (Cardini, 2014).

### 2.5 Trophic morphology in common 2D–3D shape space

Although we were able to compare the statistics that would typically be performed in a study of parallelism with either 2D or 3D landmarks, data in the separate shape spaces could not be compared directly to describe how shape was being distorted in 2D projections. That is, calculation of the angle between a vector defined by 2D landmarks and 3D landmarks is necessary to describe how the shape changes along their trajectories through morphospace differ, but is impossible if the vectors exist in two distinct spaces. On the other hand, when raw landmark sets are simply placed in the same shape space, they occupy distinct regions of it because the primary axis of variation between shapes is the difference in depth between 2D and 3D shapes. To address this problem, we used a modified version of the procedure described by Cardini (2014) and Cardini and Chiappelli (2020) of taking residuals of consensus shapes in 2D and 3D landmark sets and conducting Procrustes superimposition on both residual datasets together (see Appendix S1 Section 4 for details). Using residuals has the effect of centering both 2D and 3D landmarks sets about the consensus shapes in each dimension, rather than segregating 2D and 3D landmarks of specimens to different regions of the shape space. Instead of consensus (mean) shape residuals, we used centroid size residuals for this procedure to remove variation resulting from common allometry. Tangent space PCAs were used to visualize relationship between individuals' 2D and 3D datasets in the shared shape space.

In the common shape space, we used ANCOVAs to test whether morphological associations with diet affected the degree to which trophic levers were distorted in 2D projections. These ANCOVAs used Procrustes distances between 2D and 3D shapes of the same specimens as the dependent variable, *watershed* as an independent factor and either *diet LD* or *Simpson* as covariates. These tests would show whether there are linear relationship between the diet gradients and the disparity between 2D and 3D projections of specimens, and the strength of these relationships. Although we were able to test for distance in shape space, ANCOVAs were blind to the direction of change in shape space from 2D to 3D landmark sets. Therefore, we also conducted pairwise comparisons of angle and magnitudes as described above, using *diet LD* and *Simpson* as covariates. We also used the common shape space to compare vectors of the same watershed between 2D and 3D landmark data. We repeated these tests for the entire dataset, not grouped by watershed. In the cases of these vector analyses, an angle of 0° would mean that the trends along diet gradients, and between lakes and streams, are not distorted by the use of 2D landmark data.

### 2.6 Dimensionality of data and trophic lever function

To test how much parallelism of estimated biomechanical function is influenced by landmark dimensionality, we calculated kinematic transmission (KT) of the Op4 and Max4 linkages using the *R* package *LinkR* (Olsen, 2019; Olsen & Westneat, 2016), as well as opening and closing mandibular lever ratios (LRs). KT estimates were calculated as the ratio of output link rotation to input link rotation (Alfaro et al., 2005; Hulsey & Wainwright, 2002; Thompson et al., 2017). Essentially, both KT and LR calculations represent the efficiency of the motion that goes into a lever system in producing an output motion (Westneat, 1994). Both Op4 and Max4 KTs were calculated using an arbitrarily determined 10° of input rotation. In the Max4 linkage, the nasal link (Max2 in Figure 1) is typically defined as the coupler link (Hulsey & Wainwright, 2002; Westneat, 1990), but for purposes of analysis, we defined it as the output link. This allowed us to calculate its rotation about a fixed axis, but is not meant to imply that its elevation is more functionally important than maxillary rotation. See Appendix S1 Section 5 and Figures S2–S3 for further details and analysis.

Estimates of KT and LR were made using the same landmarks as the GM analyses, but while GM analyses used centroid size residuals of coordinates, functional estimates could not be adjusted for size in the same way because KT and LR values cannot be calculated from residuals. Because KT is many‐to‐one mapped, parallelism of KT calculations may not by accurately interpreted from KTs calculated using shape residuals of size added to consensus shapes of each linkage, either. Instead, KT and LR values were calculated from each specimen's coordinates, then regressed against Log_10_ transformed centroid sizes of entire landmark sets. The KT and LR residuals of these regressions were used in subsequent analyses as size‐corrected KT or LR.

ANCOVAs were conducted on KT and LR residuals using *watershed* as a factor and either *habitat*, *diet LD*, or *Simpson* as covariates. We used these tests to determine how parallel estimated function was with respect to diet gradients, which are presumed to be the environmental variables responsible for imposing selection on trophic function. To conduct similar analyses using all estimated biomechanical functions as dependent variables at once, we to performed PERMANOVAs, nonparametric tests with multivariate dependent variables, using the *anova.lm.rrpp* function in the *RRPP R* package (Collyer & Adams, 2018, 2019). The *anova.lm.rrpp* function uses a permutation procedure resulting in similar outputs to the *adonis* function in the *vegan* (Oksanen et al., 2019), but is able to calculate type II sums of squares (Collyer & Adams, 2018). This multivariate analysis considered biomechanical estimates as functions that work in concert to execute the process of feeding.

As with shape, we used the *pairwise* (Collyer & Adams, 2019) function with 10,000 permutations to compare angles and lengths of vectors in a space defined by the biomechanical estimates we calculated. These tests also used *diet LD* score and *Simpson* as covariates, and were run with and without watershed as a factor. Angles and magnitudes were compared between 2D kinematic models and 3D kinematic models to determine the degree to which 2D and 3D functional space vectors are parallel.

**RESULTS**

### 2.7 Diet

The diet LDA described a consistent pattern of differences in diet between lake and stream populations across all watersheds (Figure S2), with each lake population having a lower mean diet LD score than the stream population in the same watershed. It was able to assign 65.7% of individuals from which diet data were collected to the correct habitat type. Stickleback from lakes ate more zooplankton prey, including harpacticoid, calanoid, and cyclopoid copepods and *Bosmina*, *Polyphemus*, and *Daphnia* cladocerans, as well as psychomyiid and polycentropodid caddisflies. Stickleback from streams ate more empidid and chironomid dipterans, ostracods, hydracarina water mites, plecopteran, and freshwater clams. Gini–Simpson diversity ranged from 0.818–0.896.

### 2.8 Trophic morphology in separate 2D and 3D shape spaces

**FIGURE 2:**
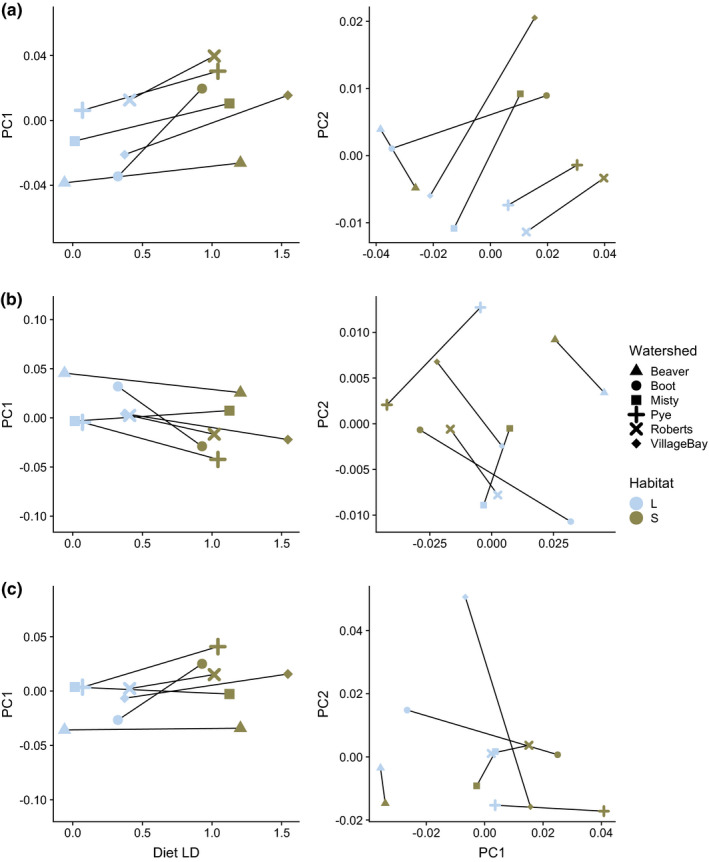
First principal components of lever shapes plotted against diet LD score are plotted in the left column. Large symbols represent population means and lines join lake and stream populations of the same watershed. Population means for each lever mechanism are shown in PC space in the right column, again with lines joining populations of the same watershed: Op4 (A; PC1%—71.1%, PC2%—10.9%), Mand (B; PC1%—73.5%, PC2% –8.5%), and Max4 (C; PC1%—47.9%, PC2%—26.2%). Tangent space PCA was conducted on landmark sets with 3D coordinates

Whether the landmarks used for shape analyses were 2D or 3D did not typically affect the statistical significance of variables in Procrustes ANOVAs. For all lever systems, variation between watersheds had significant parallel components of variation explained by *diet LD* and *habitat* (Figure 2). Contrary to expectations, the *diet LD* explained less of the variance in shape than the categorical habitat variables in most comparable models. Effect sizes did differ between 2D and 3D shapes, sometimes substantially (full results in Tables S2–S4). Our results suggest that using 3D rather than 2D landmarks slightly decreased the parallelism apparent in these populations, with diet type explaining 1%–4% less shape variance. Additionally, the main effects of *Watershed* increased in the 3D versions of tests, especially in the Op4 mechanisms, for which the R^2^ value increased from 0.177 to 0.306 in the Watershed*diet LD models (*F*_5,47_ = 3.40, *Z* = 2.44, *p* = .002 in 2D; *F*_5,47_ = 6.62, *Z* = 3.65, *p* < .001 in 3D). This difference in the variance explained between Op4 in 2D and 3D datasets can be attributed to the first Principal Component (PC) of shape in the 3D dataset, which described the distance of the linkage from the midsagittal plane, and accounted for 71.1% of Op4 shape differences between individuals.

**FIGURE 3:**
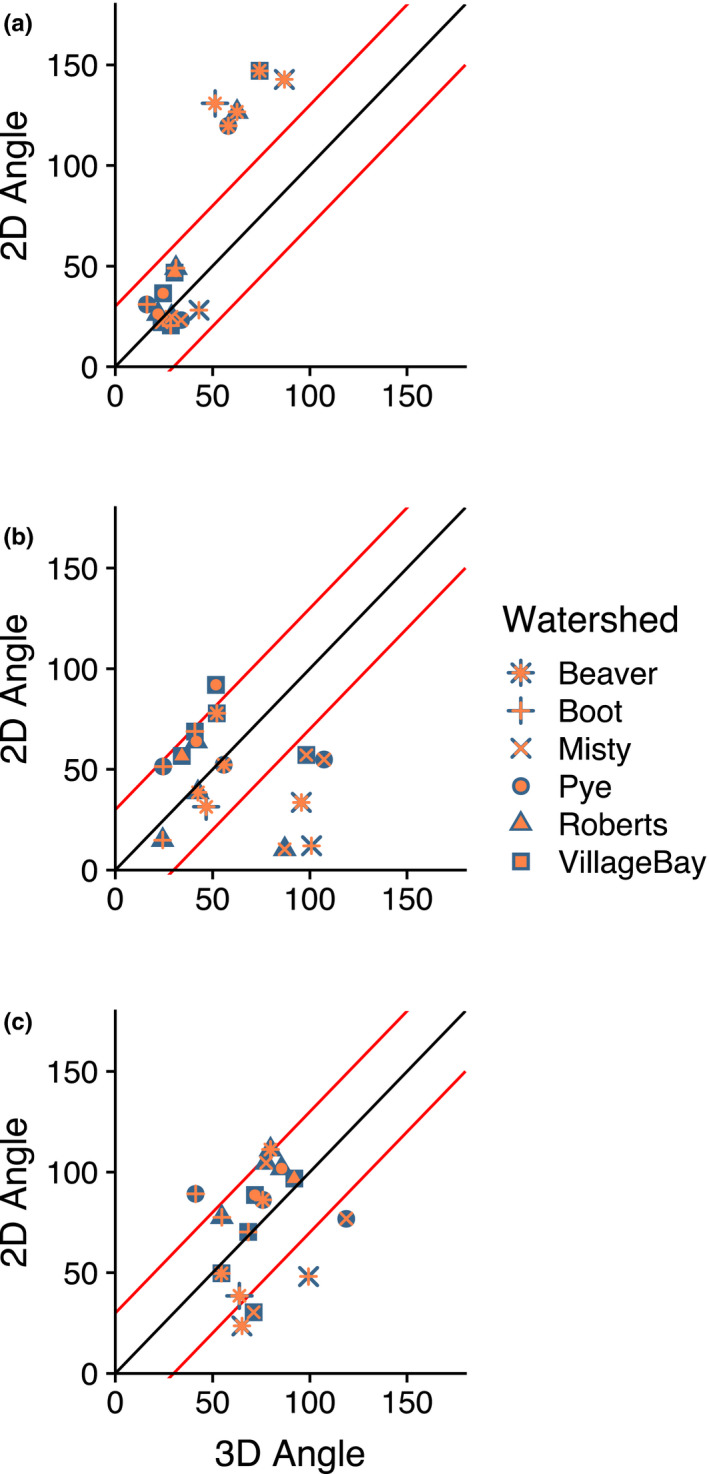
Differences in between‐watershed pairwise angles of morphospace vectors in response to Diet LD score, acquired from 2D and 3D sets of geometric morphometric landmarks. Axes indicate angle in degrees between watershed vectors, and diagonal lines indicate the difference in angle between vectors, using to 2D and 3D landmarks. The black diagonal represents no difference in angle, and the two diagonal lines on either side represent differences of 30°. Each point contains shapes representing both watersheds in the comparison, but light and dark symbols were assigned arbitrarily and do not represent any variable. Panels show data from the opercular linkage (a), mandibular lever (b), and maxillary linkage (c) datasets

Although Procrustes ANOVA results were generally similar from 2D and 3D landmark sets, cross‐watershed comparisons of vector angle and magnitude showed that the use of 3D landmarks altered interpretations of phenotypic parallelism in some cases (Figure 3, Figure S5–S6). In some comparisons, the change in angle revealed that watersheds appearing to respond in parallel to diet type from 2D data actually respond on axes that are closer to orthogonal. For instance, the comparison of the Max4 linkage between Misty and Boot watersheds went from 12.1° from the 2D data to 100.9° from 3D data, and the Roberts‐Misty comparison went from 10.3° to 87°. In the Op4 linkage, all comparisons between the Beaver watershed and other watersheds flipped from being obtuse (119.7°–147.0°) to acute (51.2°–89.6°), that is, more parallel in 3D (Figure S3). For vector magnitude, pairwise comparisons also demonstrated differences between 2D and 3D datasets (Figure S4). In particular, comparisons derived from the 3D (but not 2D) dataset revealed that the magnitude of change in Boot watershed responses to diet type was much greater in all lever systems than in the other watersheds.

Correlations between Log_10_ transformed Procrustes distances between bilaterally symmetric landmark sets and their consensus shapes revealed relatively weak correlations between 2D and 3D datasets of Op4 (*r* = .435) and Mand (*r* = .120), and a somewhat stronger correlation for Max4 datasets (*r* = .697; Figure S5). The results of Mantel tests between 2D and 3D pairwise Procrustes distance matrices showed a similar pattern (Op4: *r* = .3402, *p* = .001; Mand: *r* = .2184, *p* = .006; Max4: *r* = .7355, *p* = .001).

### 2.9 Trophic morphology in common 2D–3D shape space

**FIGURE 4:**
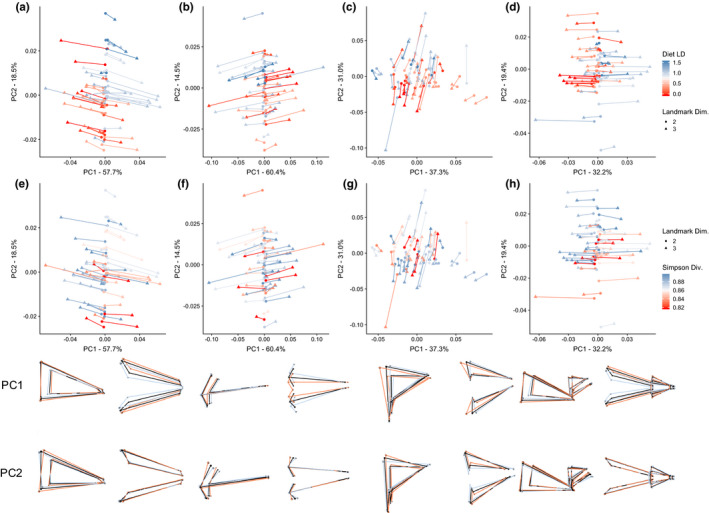
Plots of lever mechanisms and total landmark sets in common 2D‐3D shape spaces. Plots represent shape spaces of the Op4 linkage (a, e), the mandibular levers (b, f), the Max4 linkage (c, g), and the full landmark set (d, h). Color gradients for points on the plots represent values on the diet LD axis (top row) and Gini–Simpson Diversity. Lines join points representing 2D and 3D landmark sets of the same individual. Wireframe diagrams represent the PC axes of the lever mechanisms in the above plots, with blue representing PC minima, orange representing PC maxima, and black representing 3D consensus shapes. For each plot, lateral (left) and dorsal (right) views are shown. Although the plots contain shapes of both 2D and 3D landmark sets, only 3D wireframes are shown because they produce more intuitive visualizations. Wireframes show symmetrical shapes to orient the reader, but the common shape space from which the plotted data was used included only one side of each lever mechanism. Both plotted data and wireframes are adjusted for differences in shape associated with centroid size

In plots of tangent PCAs, it became evident based on the disparity between 2D and 3D shapes of the same individuals that a large amount of information was being lost in 2D projections of shape data, despite broadly similar results of Procrustes ANOVAs (Figure 4). This loss was most extreme in the Op4 linkage and mandibular lever system, from which variation in shape among 2D representations of specimens was almost entirely absent from PC1, which described 57.7% (Op4) and 60.4% (Mand) of variation in all 2D and 3D projections of shapes. The information lost in 2D projections of the Max4 linkage was less extreme, and occurred mostly in the PC2 axis. Max4 PC2 explained 31.0% of variance and mostly described the length of the fixed bar and the distance of the articular from the midline.

Although there were clear ridges to which 2D morphological projections were confined in shape space, and patterns in the direction of information loss in 2D projections related to diet type, *diet LD* had no significant effects on Procrustes distances between 2D and 3D representations of the same individuals. This was true in ANCOVAs for the lever systems, and for the landmark set including all landmarks (*p* > .39 for all). However, *Simpson* had a statistically significant effect on the Procrustes distance between 2D and 3D Op4 linkages (*F*_1,47_ = 4.99, *p* = .03) showing populations with higher prey diversity having a larger difference in shape between 2D and 3D. The complete landmark dataset showed a marginally insignificant effect of diversity in the opposite direction (*F*_1,47_ = 3.90, *p* = .054; Table S5).

Procrustes distance ANCOVAs, however, did not take into account the direction in shape space in which 3D shapes differed from 2D shapes, so they could not differentiate between specimens that were distorted the same amount, but in opposite directions in shape space. Using 2D–3D vector correlations, we were able to determine whether landmark dimensionality had an effect on the angle or magnitude of change in response to diet type or diversity both within watersheds and over the whole dataset (complete results in Tables S6–S7 and Tables S8–S9, respectively). 2D–3D dataset comparisons within watersheds rarely detected significantly different angles or magnitudes, except in the Boot watershed. However, the minimum angle between 2D and 3D vectors of a watershed was 21.5°, and the average angle was 54.4°. The angles and magnitude differences between 2D and 3D vectors across all watersheds were significantly different for the Op4 linkage and the complete landmark sets when diet LD score was used as a covariate (for Op4 and Complete, respectively, angles: 62.8°, UCL(95%) = 42.3°, Z = 3.90, *p* = .002; 54.5°, UCL(95%) = 45.5°, Z = 2.86, *p* = .009; difference in magnitude: *d* = 0.014, UCL(95%) = 0.010, Z = 3.07, *p* = .007; *d* = 0.009, UCL(95%) = 0.007, Z = 2.68, *p* = .013). When diversity was used as a covariate instead of diet type, only the difference between 2D and 3D vector magnitudes in Op4 was significantly different (*d* = 0.299, UCL(95%) = 0.251, Z = 2.47, *p* = .025). However, angles between 2D and 3D vectors still deviated from the expectation of parallelism, ranging from 41.2° to 76.4°.

### 2.10 Dimensionality of data and trophic lever function

**FIGURE 5:**
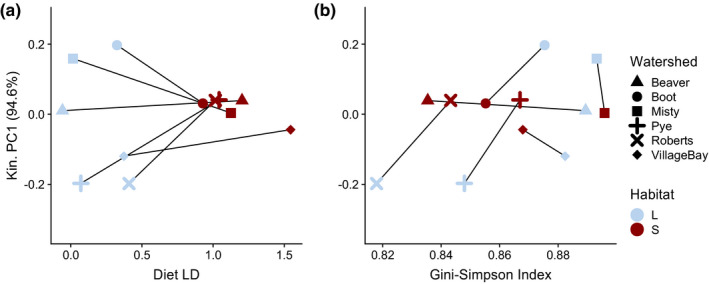
Kinematic PC1 values from a PCA on centroid size residuals of all four biomechanical variables that were calculated. Op4 KT was calculated using a kinematic model with an axis of rotation at the input joint oriented at a 30° angle from the Z axis. Max4 KT was calculated using a kinematic model with an axis of rotation at the output joint oriented at a 20° angle from the *Z* axis. All biomechanical variables had similar loadings onto PC1 (0.47–0.57)

ANCOVAs of Op4 KT, Max4 KT, and mandibular lever opening and closing LRs for 2D and 3D kinematic models showed effects of diet type (Figure S9) and diversity that were similar between 2D and 3D landmark datasets. Op4 KTs, did show consistent, and statistically significant, main effects of *diet LD* (2D: *F*_1,47_ = 5.3, *p* = .026; 3D: *F*_1,47_ = 6.2, *p* = .016), but there were also main and interaction effects of watershed (2D: *F*_5,47_ = 10.2, *p* = 1.2 × 10^−6^; 3D: *F*_5,47_ = 9.45, *p* = 2.8 × 10^−6^ for *Watershed*; 2D: *F*_5,47_ = 5.80, *p* = .0003; 3D: *F*_5,47_ = 5.70, *p* = .0003 for Watershed*diet LD) that resulted in convergence of residual Op4 KTs toward the positive (more stream‐like) end of the diet LD axis. Diet type had no significant effects on Max4 KT or either LR (2D: *F*_1,47_ = 0.16–2.0, *p* ≥ .17 in all cases; 3D: *F*_1,47_ = 0.38–2.3, *p* ≥ .14 in all cases). However, these ANCOVAs always showed significant effects of *Simpson* that resulted in higher KTs and opening and closing LRs in more diverse populations (see Tables S10–S12 for complete results). Results of corresponding PERMANOVAs that included all four kinematic variables were similarly consistent, but none showed statistically significant main effects of *diet LD*, although they did show responses of moderate effect size (2D: *R* ^2^ = .015, *F*_1,47_ = 2.0, *Z* = 0.99, *p* = .169; 3D: *R* ^2^ = .020, *F*_1,47_ = 2.5, *Z* = 1.14, *p* = .124; complete results in Table S13). Like Max4 KT and the mandibular LRs, PERMANOVAs did show positive main effects of diet diversity that were slightly larger than main effects of *Watershed* and slightly smaller than Watershed* Simpson interactions (2D: *R* ^2^ = .052, *F*_1,47_ = 6.8, *Z* = 1.93, *p* = .011; 3D: *R* ^2^ = .049, *F*_1,47_ = 6.2, *Z* = 1.85, *p* = .014). Together, these kinematic variables show multivariate functional convergence in populations with more stream‐like diets, and increasing values with increasing diversity (Figure 5). However, this trend is driven mostly by variation in kinematic estimates among the lake sites, with stream site estimates being similar.

*Diet LD* score was a stronger predictor of KT or LR disparity than was diversity (Appendix S1 Section 5, Figure S9, and Table S14). Disparity of both opening and closing LRs were significantly affected by diet LD score (Opening: *F*_1,47_ = 6.1, *p* = .017; Closing: *F*_1,47_ = 11.7, *p* = .001, respectively), with both being slightly higher at sites with more stream‐like diets. Diet diversity had no main effects, however. Disparity PERMANOVAs showed clear effects of diet type on kinematic variables in habitat*diet LD models (*R* ^2^ = .102, *F*_1,47_ = 7.1, Z = 2.18, *p* = .004), but no significant main effects of diet diversity (Table S15). This result means that morphology associated with diet type does influence the degree to which functional estimates differ between 2D and 3D‐landmarked datasets.

When pairwise comparisons were conducted on vectors describing the interactions between kinematic model dimensions and watershed, produced from a Watershed*model dimensions*diet LD model in *lm.rrpp*, no 2D‐3D comparisons of the same watersheds resulted in angles significantly different from zero (all angles ≤ 5.5°, *p* ≥ .95 in all cases; Table S16). The same was true when *Simpson* was used as a covariate (all angles ≤ 5.5°, *p* ≥ .9 in all cases), rather than diet type. Vector magnitudes were never significantly different between 2D and 3D models for either covariate (*p* > .86 in all cases). When *watershed* was excluded from the lm.rrpp models and vectors from kinematic variables including all specimens were compared, there were still no significant differences between 2D and 3D kinematic models (angle = 19.5° and 0.5° with *diet* LD and *Simpson*, respectively as covariates, *p* > .81 in all cases; Table S16). See Appendix S1 Section 5, Figure S8, and Tables S17–S24 for comparisons of additional kinematic models.

**DISCUSSION**

### 2.11 Information loss in 2D projections

We found that 2D projections of inherently 3D trophic structures resulted in lost information regarding the major components of shape variation, and this loss influences our expectations about how morphology diverges in parallel across diet gradients. For example, plots of trophic levers in common shape spaces (Figure 4) revealed that, in each of the three lever systems, and also in a combined dataset including all their landmarks, one of the first two PCs always contained most of the information about structure width (the Z dimension). That is, the primary or secondary axis of morphological variation related to trophic levers included a major contribution from the very dimension that is absent from their calculation when using data from 2D projections. Fortunately, the PC axes that included substantial contributions from 2D shapes did generally confirm results of lake‐stream divergence from previous geometric morphometric (GM) studies of stickleback (Deagle et al., 2012; Oke et al., 2016; Ravinet et al., 2013); that is, lake fish have longer and shallower skulls relative to stream fish. 3D GM reveals that the skulls of lake fish are also narrower, which is consistent with the association of increased epaxial width and suction index with more benthic feeding behavior (McGee et al., 2013). In short, previous 2D interpretations of lake‐stream divergence generally remain valid when considered in 3D space—but those previous interpretations are lacking in the context of more complete 3D data.

### 2.12 Interpretation of morphological parallelism

Previous work on stickleback from Vancouver Island lake‐stream populations reported that most morphological traits exhibit stronger nonparallel (habitat x watershed interactions) than parallel (habitat main effect) components of evolution, with the exceptions of gill raker length and body depth (Stuart et al., 2017). However, the parallelism of these traits is robust enough that it has been confirmed repeatedly. For example, Oke et al. (2016) found that the ratio of parallel to nonparallel effects was approximately 1.3 for body shape of both wild‐caught and common‐garden fish from three of the watersheds (Misty, Roberts, and Boot) included in the present study and Stuart et al. (2017). Using all six of the watersheds that we also studied here, Kaeuffer et al. (2012) found a similar effect size ratio (1.13) for parallel to nonparallel effects on 2D body shape. We found an average parallel/ nonparallel ratio of 0.85 for 3D lever system shapes in response to diet type (Table 2) and of 1.45 in response to categorical habitat variables. The Opercular four‐bar (Op4) linkage was most parallel of the lever systems in 3D shape analysis, whether considered as a response to diet type or habitat.

Although the parallelism of functional system shapes in 3D does not obviously diverge from the effects on body shape described in previous 2D work, the specifics of the effects we calculated were quite different between 2D and 3D representations. Most obviously, 2D shape spaces were not strongly correlated with their 3D counterparts (Figure 3, Figure S8). Additionally, interpretation of the most important lake‐stream shape differences changed dramatically with the incorporation of the Z dimension. In fact, in a common shape space, comparisons between 2D and 3D shape vectors (the trends through shape space) of the same watershed tended more toward orthogonal (90°) than parallel (0°), with the angles between them averaging 59° for the Op4 linkage, 57° for the mandibular levers, and 52° for the Maxillary four‐bar (Max4) linkage (Tables S5–S7). Also, the differences in magnitudes of shape vectors (the change in shape space per unit of either diet LD score or Gini–Simpson diversity of prey) in pairwise watershed comparisons in separate 2D and 3D shape spaces also changed—sometimes dramatically—with the use of 3D landmarks (Figure S4). In at least one case, differences in vector magnitude increased by a factor of 12, indicating a large difference in the amount of shape change within watersheds that is attributable to diet between 2D and 3D.

In short, 3D geometric morphometric analysis reveals that the primary axis of variation in lake‐stream stickleback head morphology is mostly described by width, a dimension that is not captured in 2D studies. This hidden variation results in weak correlation between 2D and 3D shape spaces. As a consequence, the statistical tables that are produced by researchers testing for parallelism might show apparently similar effects of habitat to those described in previous work, but these results should not be understood to mean that the same morphological patterns are being described. In this context, stickleback lake‐stream parallelism is best understood as a trend toward increasing head width in more stream‐like environments, along with the more commonly described trend toward increasing head depth relative to length. The former corresponds with an axis that describes 32.2% of the variation between individuals in shared 2D‐3D shape space of the complete landmark sets, while the latter corresponds more closely to an axis that describes 19.4% of this variation.

### 2.13 Interpretation of functional parallelism

Using 16 lake‐stream stickleback population pairs from Vancouver Island, including the six we studied here, Thompson et al. (2017) found a parallel/ nonparallel effect size ratio of 0.64 for the Opening Lever Ratio (LR), and essentially no parallelism for Op4 Kinematic Transmission (KT). More specifically, the 16 lake‐stream pairs were nearly evenly split between those with a higher KT in lake fish and those with a higher KT in stream fish. In contrast, we here found that Op4 KT was the most parallel of our functional estimates with respect to both habitat type and diet type, being typically higher in stream populations, and not differing greatly between the 2D and 3D calculations (Table 3 Figure S10). Both LRs and Max4 KT were approximately three times as parallel as Op4 KT with respect to diet diversity, rather than type. Interestingly, the multivariate effect of the diet gradients showed large functional differences between sites with lake‐like diets, but functional convergence among those with stream‐like diets. We suggest this higher variance among lake populations reflects adaptation along the benthic‐limnetic axis of stickleback morphology (McGee et al., 2013; Walker, 1997; Willacker et al., 2010), or may be an adaptation to specialist or generalist feeding, as suggested by the increase in most biomechanical estimates with increasing prey diversity.

**TABLE 3:** Parallel/nonparallel effect size ratios of diet type, habitat, and diversity on KT or LR in 2D and 3D ANCOVAs

Biomechanical Model	2D Diet LD	3D Diet LD	2D Habitat	3D Habitat	2D Simpson Div.	3D Simpson Div.
Op4	0.27	0.31	0.36	0.42	0.10	0.09
Mand Opening	0.08	0.10	0.11	0.13	0.31	0.29
Mand Closing	0.01	0.02	0.01	0.02	0.34	0.33
Max4	0.04	0.07	0.05	0.09	0.33	0.30

Note

Effect sizes used for ratios are partial Eta^2^ values (SS_effect_/[SS_effect_ + SS_error_]).

We did find some interactions between diet and landmark dimensionality, suggesting that the incorporation of the width dimension did sometimes slightly change interpretations of divergence in function. For example, the difference between 3D and 2D opening and closing LRs decreases, then becomes negative, in increasingly stream‐like fish. However, these few effects resulted in only negligible absolute differences in KT and LR values (Figure S10). Nevertheless, it is possible that for more dorso‐ventrally compressed species (i.e., those that are wider relative to their body depth), misleading interpretations of evolutionary change in function might arise from the use of 2D landmark data, depending on the biomechanical assumptions made. This may also be true for functions in which lateral expansion plays an important role, as in the abduction of the opercles during expansion of the buccal cavity.

Parallelism of trophic function was much less sensitive to dimensionality than was parallelism of trophic lever shape (see the previous section). Between 2D and 3D datasets, parallel/ nonparallel effect ratios for function do not change by more than 0.08, and usually much less (Table 4, Table S25). Meanwhile, parallel/ nonparallel effect ratios for morphology changed by 0.14–0.38, and were even larger when habitat categories were used as independent variables instead of diet type (Table 4). As might be expected of many‐to‐one mapped traits, estimated kinematic functions differed from shape in the patterns of parallelism that they exhibited, but contrary to expectations of many‐to‐one mapped traits, this pattern did not necessarily show stronger associations of functional traits with environmental gradients. It is possible that other estimates of trophic function, like suction index or jaw protrusion, are more correlated with the components of shape that were sensitive to dimensionality in our analysis. It is also possible, and perhaps likely, that these components of shape are associated with environmental or diet variables that were not captured in our analyses.

**TABLE 4:** Parallel/nonparallel effect size ratios of diet type and habitat on shape in 2D and 3D Procrustes ANOVAs

Lever System	Diet type		Habitat	
	2D	3D	2D	3D
Op4	1.04	1.42	1.36	2.63
Mand	1.01	0.67	1.63	1.15
Max4	0.61	0.47	0.44	0.56

Note

Effect sizes used for ratios are *R* ^2^ values.

## 3 CONCLUSIONS

Our finding that important conclusions change when 3D shapes are projected into 2D space reinforces other recent work demonstrating the importance of 3D morphometrics for understanding evolutionary changes in shape (Álvarez & Perez, 2013; Buser et al., 2018; McWhinnie & Parsons, 2019; Santana et al., 2019). However, our study is perhaps the first to show explicitly how this misrepresentation of shape might contribute to misinterpretations of parallel evolution. In particular, we found that trophic evolution associated with head width—which is necessarily overlooked in 2D geometric morphometric studies—is important in lake‐stream stickleback divergence. In short, with 2D geometric morphometric studies, researchers could mistakenly conclude strong parallel evolution is present when in fact it is weak, or vice‐versa, because they have failed to capture some of the most important aspects of morphological variation. In some cases, a partial solution to this problem when it is not possible to collect 3D landmark data may be to combine 2D GM data with functionally relevant linear measurements along the Z dimension, such as gape or epaxial width. Because width measurements are orthogonal to the XY plane of the 2D landmark coordinates, they do not suffer from the same measurement redundancy issues as the earlier linear measurement‐based morphometric methods as described by Zelditch et al. (2012b).

In contrast to this finding of important consequences of the third dimension on interpretations about morphological evolution, effects on interpretations about functional evolution were much smaller; 3D and 2D kinematic models resulted in very similar values of functional values. However, this conclusion comes with several important caveats. First, we had to make assumptions regarding the rotational axes of joints that are probably conservative in that they likely do not reflect the full range of motion in stickleback. Second, it was necessary to account for allometry by using different methods for shape than for functional analyses, which might have contributed to the difference in the effect of dimensionality between shape and kinematic data. Finally, although the kinematic differences between the rotational axis orientations used in our linkage models were negligible, different axis orientations resulted in differences in estimated effects of diet on the 2D–3D disparities in estimated function. Therefore, for morphological analyses, 3D GM should be used whenever possible, and for functional analyses researchers should carefully consider their own systems or conduct small‐scale pilot studies before assuming that trophic adaptations will result in patterns that are equally detectable from 2D and 3D landmarks.

It will be interesting to see how methods develop in the future to strengthen our understandings of morphological and functional adaptations to the environment through function. The shape trajectory approach described by Martinez and Wainwright (2019), for example, in which shape change is tracked through morphospace throughout the course of a kinematic process, could prove informative for determining which aspects of 3D shape are important for capturing functional divergence. This approach might be especially useful where many‐to‐one mapped functions, like multi‐bar linkages, are involved because they typically use non‐coplanar kinematics. In these cases, functional outputs are expected to be constrained by selection more than morphology, which can be more free to evolve as consequences of nonadaptive forces like drift or genetic bottlenecks without necessarily resulting in functional changes.

Although collection of 3D landmark data by microscribe and CT scanning remains costly, especially for the sample sizes required to make comparisons of multiple populations, the recent development of tools facilitating stereo‐photographic data collection has made it a viable and cheaper alternative in many cases (Olsen & Westneat, 2015). As collection of 3D morphometric data becomes more accessible, it should become the standard in most research contexts except in some cases requiring the use of live specimens or remote field work. This need for 3D data is likely to be especially true in intraspecific studies—in which morphological variation is often more subtle than between species that are not closely related—and studies in which the importance of the data that will be lost to 2D projection is unknown.

## CONFLICTS OF INTEREST

The authors have no conflicts of interest to declare.

## AUTHOR CONTRIBUTION

**Grant Emerson Haines:** Conceptualization (lead); Data curation (lead); Formal analysis (lead); Investigation (lead); Methodology (lead); Visualization (lead); Writing‐original draft (lead); Writing‐review & editing (equal). **Yoel E. Stuart:** Investigation (equal); Project administration (supporting); Supervision (equal); Writing‐review & editing (supporting). **Dieta Hanson:** Data curation (supporting); Investigation (supporting); Writing‐review & editing (supporting). **Tania Tasneem:** Data curation (supporting); Investigation (supporting); Writing‐review & editing (supporting). **Daniel I. Bolnick:** Funding acquisition (equal); Writing‐review & editing (supporting). **Hans Larsson:** Methodology (supporting); Resources (equal); Supervision (equal); Writing‐review & editing (supporting). **Andrew Hendry:** Conceptualization (equal); Funding acquisition (equal); Methodology (supporting); Project administration (lead); Resources (lead); Supervision (equal); Visualization (supporting); Writing‐original draft (supporting); Writing‐review & editing (equal).

## Supporting information

Appendix S1

## Data Availability

Data are available on Dryad at https://doi.org/10.5061/dryad.pg4f4qrmk, and code is available on GitHub at https://github.com/ghaines3/2Dv3D‐parallel.
