# Supplementary material for: Adding the third dimension to studies of parallel evolution of morphology and function: An exploration based on parapatric lake‐stream stickleback
Source: Ecol Evol. 2020 Nov 17;10(23):13297–311. doi: 10.1002/ece3.6929 (PMC7713967; doi:10.1002/ece3.6929)
Supplement: Supplementary file 1 — Appendix S1 [file ECE3-10-13297-s001.docx]

**Appendix 1**

| **Table S1.** Specimens requiring interpolation of landmarks | | |
| --- | --- | --- |
| Specimen | Population | Landmark(s) interpolated |
| S130470 | Beaver Stream | R_Op-Hy |
| S130423 | Boot Stream | R_Dent, L_Dent |
| S130537 | Misty Stream | R_Dent, L_Dent |
| S131084 | Village Bay Stream | R_vOp, R_Dent, L_Dent |

**§1.** **Data preparation**

To directly compare 2D and 3D sets of landmarks of the same specimens, there are two possible methodological approaches that can be used to acquire landmark datasets. In the first, 2D and 3D representations of a specimen (i.e., a photo or X-ray image, and a surface mesh file) are landmarked separately. 2D XY coordinates are produced from the 2D representation, and 3D XYZ coordinates are produced from the surface mesh ([Buser et al., 2018](#_ENREF_9), [e.g., Cardini, 2014](#_ENREF_11)). In the second methodological approach, only a 3D representation of the specimen is created, and is landmarked once, producing a single set of 3D landmarks. To produce the 2D landmark dataset, all specimens are rotated to a standardized orientation in coordinate space, such that the desired plane into which the 2D landmarks are to be projected is situated along an XY plane. The Z coordinates are then dropped, for analysis of 2D landmarks on their own, or set equal to zero, for analysis in a common 2D-3D shape space ([Santana et al., 2019](#_ENREF_53)).

The benefit of the second set of procedures over the first is that, when 2D and 3D landmarks are placed separately, two steps are included which introduce error. The first step is the capture of two separate representations of the specimen: a 3D representation that is expected to faithfully represent the specimen within the limits of the machinery being used (most obviously resolution), and a 2D representation – typically a photograph – that can only faithfully represent the plane that is perpendicular to the line of sight, and thus is sensitive to the orientation of the specimen. The second source of error is the landmarking itself. Each time a specimen is landmarked, the coordinates of the landmarks will differ by some (hopefully small) amount. All downstream analysis of this process that compares 2D with 3D coordinates, tests not only the effect of dimensionality on shape, but also the effects of the error introduced by image capture and separate landmarking.

For the orientation step, we used the plane of best fit through five landmarks to define the midsagittal plane ([Katz, 2017](#_ENREF_30)): the suture of the frontal bones, the anterior tip of the first copula, the anterior dorsal edge of the foramen for the carotid artery, the dorsal-most edge of the 1^st^ vertebra-basioccipital joint, and the dorsal most point of the supra-occipital crest. In three specimens, the vertebra-basioccipital joint fell outside the field of view of the scanner, so the centroid of the other four landmarks was used as the fifth point to define the mid-sagittal plane for these specimens. While other methods can be used to define the midsagittal plane, these require either the assumption that one side of the specimen is intact, or any distortion was uniform across the specimen ([Gunz et al., 2009](#_ENREF_21)), and in our case, tests and adjustments for asymmetry needed to follow this step to ensure 2D and 3D data was generated from the same projection before any such processing.

**§2. Diet Type and Diversity**

In addition to an LDA, we applied a Principal Components Analysis (PCA) to the Hellinger transformed diet data, using the *rda* function in the R package *vegan* ([Oksanen et al., 2019](#_ENREF_42)). For the diet LDA and PCA, each fish from which gut content data was collected was coded as either lake or stream. No information about watershed was included in either analysis because the objective was to produce an axis that best described the difference in diet between lake and stream fish (LDA), or the axes that best described variation in diet between individuals (PCA).

In addition to the Gini-Simpson diversity index, we calculated the Shannon index of the stickleback gut contents, also using the *diversity* function of *vegan* ([Oksanen et al., 2019](#_ENREF_42)). Shannon and Gini-Simpson diversity were calculated using the sums of recorded diet data of all individuals within each population to describe stickleback prey diversity. This produced a single value for both diversity indices to represent each population. Because gut contents were recorded as proportions of total individual diet, diversity indices calculated in this way cannot be said to represent the prey community diversity of a population (this would require the sum of counts, rather than proportions, of prey types), nor the mean prey diversity of individuals in a population (which would require the diet diversity of each individual to be averaged). Instead, they reflect diversity of the mean diet within a population.

Individuals’ PC scores were extremely variable between specimens, and no PC explained more than 16.5% of variance in gut contents among individuals. The first five PCs explained less than 50% of variance in diet. Shannon diversity values ranged from 2.17-2.57, and was highly correlated with Gini-Simpson diversity (Pearson’s r = 0.935). For these reasons, *dietPC1* and *Shannon* were used in the initial Procrustes ANOVAs (Tables S2-S4), but were excluded as an explanatory variable in subsequent analyses of shape and function.

**§3. Trophic Morphology in Separate 2D and 3D Shape Spaces**

Previous studies of the Vancouver Island parapatric stickleback populations have used the term “parallel” to refer to the relationship between habitat type (i.e., discrete lake and stream categories) and phenotypes, and used environmental variables or evolutionary history based on genotypes to explain deviation from parallelism defined by these categories ([Stuart et al., 2017](#_ENREF_56), [Kaeuffer et al., 2012](#_ENREF_29), [Paccard et al., 2019](#_ENREF_47)). While dividing habitats into these two discrete categories simplifies a large number of variables into a single factor, making interpretation of analyses much more straightforward, framing parallelism as a response to these categories is less precise than framing it as a response to the environmental gradients that these habitats describe. Thus, it is possible that parallel adaptation to an environmental gradient can appear nonparallel with respect to habitat types, or even antiparallel if environmental variation between sites of the same habitat type is high.

In this study we use the terms parallelism and non-parallelism to refer primarily to responses to diet gradients. For our purposes this also has the advantage of excluding the components of habitat type that are not directly related to diet, but for which habitat type also serves as a proxy (e.g., flow rate, structural complexity, proportion of habitat consisting of littoral area, predator communities, dissolved oxygen concentration, etc.), and which are not expected to be strongly associated with trophic lever evolution. Furthermore, one of the diet gradients used in this study (diversity) is not well represented by the simple lake/stream categories.

**§4. Trophic Morphology in Common 2D - 3D Shape Space**

Residuals of centroid size were used for this procedure to remove variation resulting from common allometry, rather than residuals of the mean specimens as in Cardini and Cardini & Chiappelli ([2020](#_ENREF_12), [2014](#_ENREF_11)) because there was a significant common effect of allometry on some of our datasets, and we wished to exclude allometric change in shape from analyses. Specimens were aligned using Procrustes superimposition on both sides of each landmark set, but because left and right sides of each specimen were mirror images of each other in 3D, and were identical in 2D, only one side of each landmark set was used in subsequent statistics to avoid overfitting.

**§5. Dimensionality of Data and Trophic Lever Function**

KT was calculated using the formula used by Hulsey & Wainwright ([2002](#_ENREF_25)), Alfaro et al. ([2005](#_ENREF_4)), and Thompson et al. ([2017](#_ENREF_58)), which is a ratio of rotation of the output link to rotation of the input link, rather than the instantaneous KT calculation used by Olsen & Westneat ([2016](#_ENREF_46)). The former version of the KT calculation is cumulative, and as a consequence is less sensitive to starting angles of lever bars, which could not be adjusted without assuming that these starting angles do not differ between populations. Lever ratios are typically measured using the lengths of input and output lever arms as measured from the fulcrum, but this only provides an estimate of mechanical advantage when they describe a planar system ([Lujan and Armbruster, 2012](#_ENREF_36)). Instead we instead used the distances between lever arm lengths and the axis of rotation to calculate this ratio, which differs from the former calculation when the lever bars are not orthogonal to the axis of rotation.

In the opercular four-bar, the input link was rotated in the posterior direction, whereas the input link of the maxillary four-bar was rotated anteriorly. Only input and output joints in all lever systems were assumed to be revolute joints with a single degree of freedom (DoF), meaning they can only rotate in a single fixed plane. For the articular-quadrate joint, which is part of all three lever systems, this assumption is supported by both the morphology of the joint ([Anker, 1974](#_ENREF_6)) and modeling of the opercular four-bar linkage in largemouth bass ([Olsen et al., 2017](#_ENREF_44)). The joint of the lateral ethmoid and lacrimal at the dorsal end of the maxillary four-bar linkage’s fixed link was also considered to be a revolute joint, as substantial abduction of the anterior ends of the maxillae are laterally constrained by the dorsal intermaxillary ligament ([Anker, 1974](#_ENREF_6), [Westneat, 1990](#_ENREF_62)). Finally, while the ball-and-socket morphology (Figure S6) and modelling of the opercular-hyomandibular joint support a 3 DoF spherical joint, it was necessary to define it as revolute joint to set the direction of input rotation. Joints not modeled as revolute joints were modeled as spherical (3 DoF) joints.

Although for simplicity’s sake, only planar 2D kinematic models and 3D kinematic models in which the rotational axes of revolute joints were parallel to the Z axis were discussed in the main text of this paper, Op4 and Max4 KT calculations were made using several arrangements of axis orientations. Although rotation about angled axes pulls joints modeled from 2D landmarks out of the XY plane, angled axes were applied in kinematic models using both 2D and 3D landmark data for purposes of comparison. For both the four-bar linkages, three kinematic models were used with different axes of rotation for relevant joints. For the opercular-hyomandibular joint of the Op4 linkage, axis orientations included one axis parallel to the global Z axis, and two others orthogonal to the Y axis, but angled anteriorly at 15º and 30º relative to the Z axis such that the input link abducted with rotation (Figure S7). Similarly, the axis orientations of the maxillary linkage’s output joint were set parallel to the Z axis, and tilted 10º and 20º medially along the YZ plane. The axis of the articular-quadrate joint, the only other one DoF joint, was set parallel to the Z axis in all kinematic models. Analyses of function were performed using these alternate versions of kinematic models in addition to the simpler kinematic models with rotational axes of revolute joints, and results of statistical tests from alternate model versions are included in the Appendix 2 tables.

Results of KT and LR ANCOVAs for 2D and 3D kinematic model versions with alternate rotational axis orientations showed effects of diet type and diversity that were similar between 2D and 3D landmark datasets to the kinematic models with fixed rotational axis orientations parallel to the Z axis (Tables S10-S11, Figure S9).

In addition to the ANCOVAs and PERMANOVAs describing the effects of diet variables on kinematic residuals, ANCOVAs and PERMANOVAs were conducted using the disparities between biomechanical outputs in 2D and 3D within and between kinematic model versions (combinations of landmark dimensionality and rotational axis orientations). We used these tests to determine whether diet type or diversity influenced the degree to which calculated biomechanical values differed between 2D and 3D kinematic models (Tables S14-S15). The vector analyses described in the text comparing kinematic calculations between 2D and 3D kinematic models (Table S16) were also performed to compare all 2D and 3D kinematic model versions with the angled axes of rotation (Tables S17-S20).

In ANCOVAs using disparity between calculated biomechanical variables in 2D and 3D, results were highly sensitive to the versions of the 2D and 3D kinematic models being compared (Table S13). Occasionally, diet type effects reversed in response to differently angled joint axes. For instance, in comparisons between 2D and 3D kinematic models with axes angled in the same way, when *diet LD* increased, Op4 KT disparity also increased in the 0º comparison (F = 13.2, p = 0.0007), decreased in the 30º comparison (F = 12.2, p = 0.001), and showed no main effect in the 15º comparison (F = 0.8, p = 0.372). While sometimes statistically significant, these effects were far too small to be biologically significant (Figure S10). However, they do suggest that the morphologies associated with different ecotypes may affect how much kinematic variables differ between 2D and 3D calculations in other systems in a way that influences interpretations about their biology.

When pairwise comparisons are conducted on vectors describing the interactions between watershed and alternate kinematic model versions, produced from a *Watershed*model version*diet LD* model in *lm.rrpp*, no 2D-3D comparisons of the same watersheds resulted in angles significantly different from zero or significant differences in vector magnitude (all angles < 8.2º, p > 0.68 in all cases; all distances < 0.023, p > 0.85 in all cases; Tables S17-S20, Figures S7 and S10).

**Appendix 2**

**Statistical Tables**

| **Table S2.** Procrustes ANOVAs of the opercular four-bar linkage in separate shape spaces for 2D (left half of table) and 3D (right half of table) landmark datasets. Watershed * dietLD * simpson models were truncated by QR decomposition. Statistically significant effects are italicized. Significance codes: (.) 0.1, (*) 0.05, (**) 0.01 | | | | | | | | | | | | | |
| --- | --- | --- | --- | --- | --- | --- | --- | --- | --- | --- | --- | --- | --- |
|  |  | **Op4 2D** |  |  |  |  |  | **Op4 3D** |  |  |  |  |  |
| **Model** | **Df** | **SS** | **MS** | **Rsq** | **F** | **Z** | **Pr(>SS)** | **SS** | **MS** | **Rsq** | **F** | **Z** | **Pr(>SS)** |
| **watershed * habitat** |  |  |  |  |  |  |  |  |  |  |  |  |  |
| *watershed* | *5* | *0.0034605* | *0.0006921* | *0.17852* | *3.4269* | *2.8171* | *0.002 *** | *0.025447* | *0.0050895* | *0.31071* | *6.7172* | *4.5685* | *0.001 *** |
| *habitat* | *1* | *0.0036763* | *0.0036763* | *0.18965* | *18.2029* | *3.2552* | *0.001 *** | *0.014709* | *0.0147094* | *0.17960* | *19.4139* | *3.6664* | *0.001 *** |
| *watershed:habitat* | *5* | *0.0026999* | *0.0005400* | *0.13928* | *2.6737* | *2.3656* | *0.006 *** | 0.005583 | 0.0011165 | 0.06816 | 1.4736 | 1.0599 | 0.145 |
| Residuals | 47 | 0.0094922 | 0.0002020 | 0.48968 |  |  |  | 0.035611 | 0.0007577 | 0.43480 |  |  |  |
| **watershed * dietLD1** |  |  |  |  |  |  |  |  |  |  |  |  |  |
| *watershed* | *5* | *0.0034337* | *0.0006867* | *0.17714* | *3.4003* | *2.8154* | *0.001 *** | *0.025068* | *0.0050137* | *0.30608* | *6.6172* | *4.4547* | *0.001 *** |
| *dietLD1* | *1* | *0.0032534* | *0.0032534* | *0.16783* | *16.1089* | *3.1183* | *0.001 *** | *0.011921* | *0.0119208* | *0.14555* | *15.7334* | *3.4661* | *0.001 *** |
| *watershed:dietLD1* | *5* | *0.0031228* | *0.0006246* | *0.16110* | *3.0925* | *2.6462* | *0.003 *** | *0.008371* | *0.0016743* | *0.10221* | *2.2098* | *1.9751* | *0.018 ** |
| Residuals | 47 | 0.0094922 | 0.0002020 | 0.48968 |  |  |  | 0.035611 | 0.0007577 | 0.43480 |  |  |  |
| **watershed * dietPC1** |  |  |  |  |  |  |  |  |  |  |  |  |  |
| watershed | 5 | *0.0024920* | *0.00049840* | *0.12856* | *2.4678* | *2.2508* | *0.002 ** | *0.025286* | *0.0050573* | *0.30874* | *6.6748* | *4.2554* | *0.001 *** |
| dietPC1 | 1 | 0.0001315 | 0.00013151 | 0.00678 | 0.6512 | -0.0104 | 0.551 | 0.001431 | 0.0014312 | 0.01747 | 1.8890 | 1.0666 | 0.135 |
| *watershed:dietPC1* | *5* | *0.0062447* | *0.00124894* | *0.32215* | *6.1841* | *4.1520* | *0.001 *** | *0.018861* | *0.0037722* | *0.23029* | *4.9786* | *3.7858* | *0.001 *** |
| Residuals | 47 | 0.0094922 | 0.00020196 | 0.48968 |  |  |  | 0.035611 | 0.0007577 | 0.43480 |  |  |  |
| **watershed * shannon** |  |  |  |  |  |  |  |  |  |  |  |  |  |
| *watershed* | *5* | *0.0035177* | *0.00070355* | *0.18147* | *3.4836* | *2.9754* | *0.001 *** | *0.020992* | *0.0041985* | *0.25631* | *5.5413* | *4.0156* | *0.001 *** |
| shannon | 1 | 0.0000631 | 0.00006311 | 0.00326 | 0.3125 | -0.7300 | 0.765 | 0.001518 | 0.0015176 | 0.01853 | 2.0030 | 1.1753 | 0.116 |
| *watershed:shannon* | *5* | *0.0063131* | *0.00126262* | *0.32568* | *6.2518* | *4.1058* | *0.001 *** | *0.018775* | *0.0037549* | *0.22923* | *4.9558* | *3.7402* | *0.001 *** |
| Residuals | 47 | 0.0094922 | 0.00020196 | 0.48968 |  |  |  | 0.035611 | 0.0007577 | 0.43480 |  |  |  |
| **watershed * simpson** |  |  |  |  |  |  |  |  |  |  |  |  |  |
| *watershed* | *5* | *0.0033353* | *0.00066706* | *0.17206* | *3.3029* | *2.8938* | *0.011 ** | *0.021668* | *0.0043335* | *0.26456* | *5.7195* | *4.0279* | *0.001 *** |
| simpson | 1 | 0.0001857 | 0.00018571 | 0.00958 | 0.9195 | 0.3247 | 0.393 | 0.001091 | 0.0010909 | 0.01332 | 1.4398 | 0.8089 | 0.199 |
| *watershed:simpson* | *5* | *0.0061905* | *0.00123810* | *0.31835* | *6.1304* | *4.1178* | *0.001 *** | *0.019201* | *0.0038403* | *0.23445* | *5.0685* | *3.7298* | *0.001 *** |
| Residuals | 47 | 0.0094922 | 0.00020196 | 0.48968 |  |  |  | 0.035611 | 0.0007577 | 0.43480 |  |  |  |
| **dietLD1 * shannon** |  |  |  |  |  |  |  |  |  |  |  |  |  |
| *dietLD1* | *1* | *0.0038223* | *0.0038223* | *0.19718* | *13.7049* | *2.95312* | *0.001 *** | *0.009618* | *0.0096176* | *0.11743* | *8.5639* | *2.7318* | *0.003 *** |
| shannon | 1 | 0.0005480 | 0.0005480 | 0.02827 | 1.9647 | 1.06545 | 0.134 | *0.003290* | *0.0032904* | *0.04018* | *2.9299* | *1.6041* | *0.049 ** |
| dietLD1:shannon | 1 | 0.0001613 | 0.0001613 | 0.00832 | 0.5783 | -0.13968 | 0.588 | *0.003993* | *0.0039928* | *0.04875* | *3.5554* | *1.7126* | *0.043 ** |
| Residuals | 55 | 0.0153394 | 0.0002789 | 0.79132 |  |  |  | 0.061767 | 0.0011230 | 0.75417 |  |  |  |
| **dietLD1 * simpson** |  |  |  |  |  |  |  |  |  |  |  |  |  |
| *dietLD1* | *1* | *0.0039134* | *0.0039134* | *0.20189* | *14.3520* | *2.98910* | *0.001 *** | *0.010746* | *0.0107463* | *0.13121* | *9.6903* | *2.8496* | *0.002 *** |
| *simpson* | *1* | *0.0009442* | *0.0009442* | *0.04871* | *3.4626* | *1.67800* | *0.031 ** | 0.003317 | 0.0033171 | 0.04050 | 2.9911 | 1.6718 | 0.051 . |
| dietLD1:simpson | 1 | 0.0001073 | 0.0001073 | 0.00554 | 0.3936 | -0.55019 | 0.717 | *0.004740* | *0.0047400* | *0.05787* | *4.2742* | *1.8800* | *0.031 ** |
| Residuals | 55 | 0.0149972 | 0.0002727 | 0.77367 |  |  |  | 0.060993 | 0.0011090 | 0.74472 |  |  |  |

| **Table S3.** Procrustes ANOVAs of the mandibular lever system in separate shape spaces for 2D (left half of table) and 3D (right half of table) landmark datasets. Watershed * dietLD * simpson models were truncated by QR decomposition. Statistically significant effects are italicized. Significance codes: (.) 0.1, (*) 0.05, (**) 0.01 | | | | | | | | | | | | | |
| --- | --- | --- | --- | --- | --- | --- | --- | --- | --- | --- | --- | --- | --- |
|  |  | **Mand 2D** |  |  |  |  |  | **Mand 3D** |  |  |  |  |  |
| **Model** | **Df** | **SS** | **MS** | **Rsq** | **F** | **Z** | **Pr(>SS)** | **SS** | **MS** | **Rsq** | **F** | **Z** | **Pr(>SS)** |
| **watershed * habitat** |  |  |  |  |  |  |  |  |  |  |  |  |  |
| watershed | 5 | 0.003450 | 0.0006900 | 0.10468 | 1.4172 | 1.06459 | 0.146 | *0.024310* | *0.0048620* | *0.16271* | *2.2275* | *1.95676* | *0.023 ** |
| *habitat* | *1* | *0.004105* | *0.0041052* | *0.12456* | *8.4316* | *2.76984* | *0.001 *** | *0.011686* | *0.0116858* | *0.07822* | *5.3539* | *2.24633* | *0.012 ** |
| watershed:habitat | 5 | 0.002506 | 0.0005012 | 0.07604 | 1.0295 | 0.21108 | 0.423 | 0.010193 | 0.0020386 | 0.06823 | 0.9340 | -0.00111 | 0.497 |
| Residuals | 47 | 0.022884 | 0.0004869 | 0.69434 |  |  |  | 0.102587 | 0.0021827 | 0.68665 |  |  |  |
| **watershed * dietLD1** |  |  |  |  |  |  |  |  |  |  |  |  |  |
| watershed | 5 | 0.003739 | 0.0007478 | 0.11344 | 1.5358 | 1.28878 | 0.094 . | *0.023444* | *0.0046887* | *0.15692* | *2.1481* | *1.88794* | *0.028 ** |
| *dietLD1* | *1* | *0.003321* | *0.0033205* | *0.10075* | *6.8198* | *2.53775* | *0.001 *** | *0.008785* | *0.0087853* | *0.05880* | *4.0250* | *1.94729* | *0.027 ** |
| watershed:dietLD1 | 5 | 0.003291 | 0.0006582 | 0.09985 | 1.3518 | 0.90091 | 0.180 | 0.013094 | 0.0026187 | 0.08764 | 1.1998 | 0.55132 | 0.289 |
| Residuals | 47 | 0.022884 | 0.0004869 | 0.69434 |  |  |  | 0.102587 | 0.0021827 | 0.68665 |  |  |  |
| **watershed * dietPC1** |  |  |  |  |  |  |  |  |  |  |  |  |  |
| watershed | 5 | 0.003358 | 0.00067159 | 0.10189 | 1.3794 | 1.01955 | 0.168 | *0.026700* | *0.0053401* | *0.17871* | *2.4465* | *2.08560* | *0.019 ** |
| dietPC1 | 1 | 0.000509 | 0.00050878 | 0.01544 | 1.0450 | 0.39193 | 0.378 | 0.002751 | 0.0027508 | 0.01841 | 1.2603 | 0.65434 | 0.281 |
| *watershed:dietPC1* | *5* | *0.006103* | *0.00122053* | *0.18517* | *2.5068* | *2.56493* | *0.003 *** | 0.019128 | 0.0038256 | 0.12803 | 1.7527 | 1.39241 | 0.079 . |
| Residuals | 47 | 0.022884 | 0.00048689 | 0.69434 |  |  |  | 0.102587 | 0.0021827 | 0.68665 |  |  |  |
| **watershed * shannon** |  |  |  |  |  |  |  |  |  |  |  |  |  |
| watershed | 5 | 0.003855 | 0.00077093 | 0.11696 | 1.5834 | 1.39557 | 0.086 . | *0.023187* | *0.0046375* | *0.15520* | *2.1247* | *1.82298* | *0.031 ** |
| shannon | 1 | 0.000722 | 0.00072206 | 0.02191 | 1.4830 | 0.82347 | 0.203 | 0.002265 | 0.0022653 | 0.01516 | 1.0378 | 0.47943 | 0.325 |
| *watershed:shannon* | *5* | *0.005889* | *0.00117788* | *0.17870* | *2.4192* | *2.44228* | *0.006 *** | *0.019614* | *0.0039227* | *0.13128* | *1.7972* | *1.41896* | *0.083 .* |
| Residuals | 47 | 0.022884 | 0.00048689 | 0.69434 |  |  |  | 0.102587 | 0.0021827 | 0.68665 |  |  |  |
| **watershed * simpson** |  |  |  |  |  |  |  |  |  |  |  |  |  |
| watershed | 5 | 0.003648 | 0.00072967 | 0.11070 | 1.4986 | 1.23994 | 0.111 | *0.024688* | *0.0049376* | *0.16525* | *2.2622* | *1.93996* | *0.022 ** |
| simpson | 1 | 0.000357 | 0.00035669 | 0.01082 | 0.7326 | 0.01284 | 0.536 | 0.002086 | 0.0020857 | 0.01396 | 0.9555 | 0.38927 | 0.345 |
| *watershed:simpson* | *5* | *0.006255* | *0.00125095* | *0.18978* | *2.5693* | *2.60847* | *0.004 *** | *0.019793* | *0.0039587* | *0.13248* | *1.8137* | *1.44839* | *0.069 .* |
| Residuals | 47 | 0.022884 | 0.00048689 | 0.69434 |  |  |  | 0.102587 | 0.0021827 | 0.68665 |  |  |  |
| **dietLD1 * shannon** |  |  |  |  |  |  |  |  |  |  |  |  |  |
| *dietLD1* | *1* | *0.003032* | *0.00303233* | *0.09201* | *5.6437* | *2.38473* | *0.002 *** | *0.008199* | *0.0081992* | *0.05488* | *3.3419* | *1.69253* | *0.047 ** |
| shannon | 1 | 0.000318 | 0.00031805 | 0.00965 | 0.5919 | -0.35551 | 0.669 | 0.001935 | 0.0019354 | 0.01295 | 0.7888 | 0.17825 | 0.416 |
| dietLD1:shannon | 1 | 0.000044 | 0.00004428 | 0.00134 | 0.0824 | -2.64090 | 0.987 | 0.002247 | 0.0022474 | 0.01504 | 0.9160 | 0.29779 | 0.376 |
| Residuals | 55 | 0.029551 | 0.00053730 | 0.89664 |  |  |  | 0.134941 | 0.0024535 | 0.90321 |  |  |  |
| **dietLD1 * simpson** |  |  |  |  |  |  |  |  |  |  |  |  |  |
| *dietLD1* | *1* | *0.003220* | *0.0032199* | *0.09770* | *6.0013* | *2.43320* | *0.002 *** | *0.009282* | *0.0092817* | *0.06213* | *3.7533* | *1.80796* | *0.035 ** |
| simpson | 1 | 0.000347 | 0.0003466 | 0.01052 | 0.6459 | -0.20707 | 0.629 | 0.001338 | 0.0013376 | 0.00895 | 0.5409 | -0.23291 | 0.570 |
| dietLD1:simpson | 1 | 0.000057 | 0.0000574 | 0.00174 | 0.1070 | -2.27007 | 0.973 | 0.001774 | 0.0017740 | 0.01187 | 0.7173 | 0.05186 | 0.453 |
| Residuals | 55 | 0.029510 | 0.0005365 | 0.89538 |  |  |  | 0.136012 | 0.0024730 | 0.91038 |  |  |  |

| **Table S4.** Procrustes ANOVAs of the maxillary four-bar linkage in separate shape spaces for 2D (left half of table) and 3D (right half of table) landmark datasets. Watershed * dietLD * simpson modelw were truncated by QR decomposition. Statistically significant effects are italicized. Significance codes: (.) 0.1, (*) 0.05, (**) 0.01 | | | | | | | | | | | | | |
| --- | --- | --- | --- | --- | --- | --- | --- | --- | --- | --- | --- | --- | --- |
|  |  | **Max4 2D** |  |  |  |  |  | **Max4 3D** |  |  |  |  |  |
| **Model** | **Df** | **SS** | **MS** | **Rsq** | **F** | **Z** | **Pr(>SS)** | **SS** | **MS** | **Rsq** | **F** | **Z** | **Pr(>SS)** |
| **watershed * habitat** |  |  |  |  |  |  |  |  |  |  |  |  |  |
| *watershed* | *5* | *0.018190* | *0.0036379* | *0.19698* | *3.1316* | *3.1019* | *0.001 *** | *0.028693* | *0.0057386* | *0.20214* | *3.2969* | *3.6039* | *0.001 *** |
| *habitat* | *1* | *0.005885* | *0.0058854* | *0.06373* | *5.0663* | *2.0370* | *0.008 *** | *0.010985* | *0.0109853* | *0.07739* | *6.3112* | *2.7529* | *0.001 *** |
| *watershed:habitat* | *5* | *0.013308* | *0.0026617* | *0.14412* | *2.2913* | *2.2277* | *0.011 ** | *0.019703* | *0.0039406* | *0.13880* | *2.2639* | *2.5699* | *0.002 *** |
| Residuals | 47 | 0.054599 | 0.0011617 | 0.59126 |  |  |  | 0.081809 | 0.0017406 | 0.57633 |  |  |  |
| **watershed * dietLD1** |  |  |  |  |  |  |  |  |  |  |  |  |  |
| *watershed* | *5* | *0.021637* | *0.0043273* | *0.23431* | *3.7250* | *3.5387* | *0.001 *** | *0.031227* | *0.0062454* | *0.21999* | *3.5880* | *3.8308* | *0.001 *** |
| *dietLD1* | *1* | *0.007305* | *0.0073053* | *0.07911* | *6.2886* | *2.2634* | *0.004 *** | *0.009798* | *0.0097978* | *0.06902* | *5.6289* | *2.5939* | *0.001 *** |
| *watershed:dietLD1* | *5* | *0.011889* | *0.0023777* | *0.12874* | *2.0468* | *1.9504* | *0.022 ** | *0.020890* | *0.0041781* | *0.14717* | *2.4004* | *2.7201* | *0.002 *** |
| Residuals | 47 | 0.054599 | 0.0011617 | 0.59126 |  |  |  | 0.081809 | 0.0017406 | 0.57633 |  |  |  |
| **watershed * dietPC1** |  |  |  |  |  |  |  |  |  |  |  |  |  |
| *watershed* | *5* | *0.016865* | *0.0033729* | *0.18263* | *2.9035* | *2.87739* | *0.002 *** | *0.031305* | *0.0062609* | *0.22054* | *3.5970* | *3.6856* | *0.001 *** |
| dietPC1 | 1 | 0.001692 | 0.0016921 | 0.01832 | 1.4566 | 0.80042 | 0.227 | *0.004622* | *0.0046222* | *0.03256* | *2.6555* | *1.6235* | *0.043 ** |
| *watershed:dietPC1* | *5* | *0.017502* | *0.0035004* | *0.18953* | *3.0132* | *2.73087* | *0.004 *** | *0.026066* | *0.0052132* | *0.18363* | *2.9950* | *3.2088* | *0.001 *** |
| Residuals | 47 | 0.054599 | 0.0011617 | 0.59126 |  |  |  | 0.081809 | 0.0017406 | 0.57633 |  |  |  |
| **watershed * shannon** |  |  |  |  |  |  |  |  |  |  |  |  |  |
| *watershed* | *5* | *0.020042* | *0.0040084* | *0.21704* | *3.4505* | *3.3156* | *0.001 *** | *0.029184* | *0.0058369* | *0.20560* | *3.3533* | *3.5354* | *0.001 *** |
| shannon | 1 | 0.002678 | 0.0026781 | 0.02900 | 2.3054 | 1.2798 | 0.089 . | 0.002473 | 0.0024730 | 0.01742 | 1.4207 | 0.8173 | 0.219 |
| *watershed:shannon* | *5* | *0.016516* | *0.0033032* | *0.17885* | *2.8434* | *2.6607* | *0.005 *** | *0.028215* | *0.0056431* | *0.19877* | *3.2420* | *3.4088* | *0.001 *** |
| Residuals | 47 | 0.054599 | 0.0011617 | 0.59126 |  |  |  | 0.081809 | 0.0017406 | 0.57633 |  |  |  |
| **watershed * simpson** |  |  |  |  |  |  |  |  |  |  |  |  |  |
| *watershed* | *5* | *0.019230* | *0.0038459* | *0.20824* | *3.3107* | *3.2113* | *0.001 *** | *0.029446* | *0.0058892* | *0.20744* | *3.3834* | *3.5572* | *0.001 *** |
| simpson | 1 | 0.002439 | 0.0024389 | 0.02641 | 2.0995 | 1.1605 | 0.107 | 0.002314 | 0.0023136 | 0.01630 | 1.3292 | 0.7111 | 0.252 |
| *watershed:simpson* | *5* | *0.016755* | *0.0033510* | *0.18144* | *2.8846* | *2.6803* | *0.005 *** | *0.028375* | *0.0056749* | *0.19990* | *3.2603* | *3.4551* | *0.001 *** |
| Residuals | 47 | 0.054599 | 0.0011617 | 0.59126 |  |  |  | 0.081809 | 0.0017406 | 0.57633 |  |  |  |
| **dietLD1 * shannon** |  |  |  |  |  |  |  |  |  |  |  |  |  |
| dietLD1 | 1 | 0.003568 | 0.0035676 | 0.03863 | 2.2997 | 1.23082 | 0.115 | *0.006472* | *0.0064717* | *0.04559* | *2.7736* | *1.60256* | *0.048 ** |
| shannon | 1 | 0.000535 | 0.0005351 | 0.00579 | 0.3449 | -0.83938 | 0.804 | 0.001189 | 0.0011895 | 0.00838 | 0.5098 | -0.58886 | 0.717 |
| dietLD1:shannon | 1 | 0.002264 | 0.0022641 | 0.02452 | 1.4595 | 0.78796 | 0.226 | 0.004403 | 0.0044034 | 0.03102 | 1.8872 | 1.16325 | 0.115 |
| Residuals | 55 | 0.085325 | 0.0015514 | 0.92400 |  |  |  | 0.128333 | 0.0023333 | 0.90409 |  |  |  |
| **dietLD1 * simpson** |  |  |  |  |  |  |  |  |  |  |  |  |  |
| dietLD1 | 1 | 0.003556 | 0.0035565 | 0.03851 | 2.3190 | 1.23647 | 0.114 | *0.006823* | *0.0068230* | *0.04807* | *2.9207* | *1.67739* | *0.038 ** |
| simpson | 1 | 0.001097 | 0.0010970 | 0.01188 | 0.7153 | -0.01480 | 0.536 | 0.001120 | 0.0011196 | 0.00789 | 0.4793 | -0.68297 | 0.758 |
| dietLD1:simpson | 1 | 0.002676 | 0.0026761 | 0.02898 | 1.7449 | 0.97622 | 0.165 | 0.004323 | 0.0043231 | 0.03046 | 1.8506 | 1.14875 | 0.126 |
| Residuals | 55 | 0.084351 | 0.0015337 | 0.91345 |  |  |  | 0.128484 | 0.0023361 | 0.90515 |  |  |  |

| **Table S5.** ANCOVAs testing the effects of effects of diet type and diversity on Procrustes distance between 2D and 3D landmarks of the same specimens in a common 2D-3D shape space. Tests were performed for each lever system, as well as the dataset including landmarks for all lever systems combined (Total). ANCOVAs were not able to test the direction of the change between 2D and 3D versions of the same specimen in shape space, only the Procrustes distance between them. Significance codes: (.) 0.1, (*) 0.05, (**) 0.01 | | | | | | | | | |
| --- | --- | --- | --- | --- | --- | --- | --- | --- | --- |
|  | Sum Sq | Df | F value | Pr(>F) |  | Sum Sq | Df | F value | Pr(>F) |
| **Op4** |  |  |  |  |  |  |  |  |  |
| Watershed | 0.29609 | 5 | 1.7331 | 0.145543 | Watershed | *0.44901* | *5* | *2.6281* | *0.03555 ** |
| LD1 | 0.00098 | 1 | 0.0286 | 0.866344 | simpson | *0.17052* | *1* | *4.9905* | *0.03028 ** |
| Watershed:LD1 | *0.69375* | *5* | *4.0607* | *0.003813 *** | Watershed:simpson | *0.52421* | *5* | *3.0683* | *0.01774 ** |
| Residuals | 1.60595 | 47 |  |  | Residuals | 1.60595 | 47 |  |  |
| **Mand** |  |  |  |  |  |  |  |  |  |
| Watershed | 0.19127 | 5 | 0.8813 | 0.50107 | Watershed | 0.13312 | 5 | 0.6133 | 0.6901 |
| LD1 | 0.03213 | 1 | 0.7401 | 0.39399 | simpson | 0.11586 | 1 | 2.6692 | 0.1090 |
| Watershed:LD1 | 0.43489 | 5 | 2.0037 | 0.09541 . | Watershed:simpson | 0.35115 | 5 | 1.6179 | 0.1738 |
| Residuals | 2.04016 | 47 |  |  | Residuals | 2.04016 | 47 |  |  |
| **Max4** |  |  |  |  |  |  |  |  |  |
| Watershed | *0.55176* | *5* | *3.2662* | *0.0130 ** | Watershed | *0.46267* | *5* | *2.7388* | *0.02983 ** |
| LD1 | 0.00317 | 1 | 0.0939 | 0.7607 | simpson | 0.01518 | 1 | 0.4492 | 0.50598 |
| Watershed:LD1 | 0.20242 | 5 | 1.1982 | 0.3246 | Watershed:simpson | 0.19041 | 5 | 1.1272 | 0.35917 |
| Residuals | 1.58795 | 47 |  |  | Residuals | 1.58795 | 47 |  |  |
| **Total** |  |  |  |  |  |  |  |  |  |
| Watershed | *0.49887* | *5* | *3.8497* | *0.005264 *** | Watershed | *0.58759* | *5* | *4.5343* | *0.001869 *** |
| LD1 | 0.00515 | 1 | 0.1986 | 0.657898 | simpson | 0.10099 | 1 | 3.8966 | 0.054279 . |
| Watershed:LD1 | 0.26551 | 5 | 2.0489 | 0.088872 . | Watershed:simpson | 0.16967 | 5 | 1.3093 | 0.276306 |
| Residuals | 1.21811 | 47 |  |  | Residuals | 1.21811 | 47 |  |  |

| **Table S6.** Pairwise comparisons of shape vectors for 2D and 3D datasets of the same watersheds in a common shape space, using diet type as a covariate. Comparisons of angles are on the left, and comparisons of slope vector lengths are on the right. Confidence limits are single-tailed, with null hypotheses for comparisons being 0º, and a distance of 0 units in shape space, respectively. Comparisons were conducted for the opercular four-bar linkage (Op4), the mandibular lever system (Mand), the maxillary four-bar linkage (Max4), and the dataset including all landmarks (Total). Statistically significant comparisons are italicized. Significance codes: (.) 0.1, (*) 0.05, (**) 0.01 | | | | | | | | | |
| --- | --- | --- | --- | --- | --- | --- | --- | --- | --- |
| **Watershed*diet LD*Dim** |  |  |  |  |  |  |  |  |  |
| Comparison | r | angle | UCL (95%) | Z | Pr > angle | d | UCL (95%) | Z | Pr > d |
| **Op4** |  |  |  |  |  |  |  |  |  |
| Beaver.2:Beaver.3 | 0.43874336 | 63.97627 | 143.02164 | -1.41249953 | 0.892 | 0.0075155784 | 0.01933262 | -0.17196499 | 0.503 |
| *Boot.2:Boot.3* | *0.26689114* | *74.52064* | *58.23834* | *3.06686227* | *0.010 ** | *0.0652238493* | *0.04806249* | *3.01233519* | *0.009 *** |
| Misty.2:Misty.3 | 0.69169458 | 46.23560 | 88.95266 | -0.53284167 | 0.686 | 0.0074244480 | 0.02031441 | -0.20245038 | 0.504 |
| Pye.2:Pye.3 | 0.45691621 | 62.81171 | 116.25126 | -0.33920008 | 0.607 | 0.0119696396 | 0.02755372 | -0.02714599 | 0.453 |
| Roberts.2:Roberts.3 | 0.51955572 | 58.69755 | 87.52672 | 0.51558993 | 0.276 | 0.0191016476 | 0.03564936 | 0.41456616 | 0.293 |
| VillageBay.2:VillageBay.3 | 0.65164096 | 49.33456 | 71.46402 | 0.27813945 | 0.373 | 0.0111190624 | 0.02162381 | 0.14883209 | 0.397 |
| **Mand** |  |  |  |  |  |  |  |  |  |
| Beaver.2:Beaver.3 | 0.40347446 | 66.20444 | 123.32536 | -0.31867138 | 0.588 | 0.0120358916 | 0.02608786 | 0.30906682 | 0.300 |
| Boot.2:Boot.3 | 0.43079314 | 64.48209 | 68.02867 | 1.81944219 | 0.069 . | 0.0562904944 | 0.05684320 | 1.84610137 | 0.054 |
| Misty.2:Misty.3 | 0.77373543 | 39.30948 | 114.32794 | -1.14784033 | 0.862 | 0.0043607410 | 0.02415079 | -0.68715542 | 0.703 |
| Pye.2:Pye.3 | 0.46478249 | 62.30385 | 104.57275 | 0.26807232 | 0.343 | 0.0207203298 | 0.03843647 | 0.52673266 | 0.261 |
| Roberts.2:Roberts.3 | 0.56785927 | 55.39892 | 120.49291 | -0.44758275 | 0.626 | 0.0121155972 | 0.04769730 | -0.33962914 | 0.531 |
| VillageBay.2:VillageBay.3 | 0.61727528 | 51.88257 | 102.98313 | -0.27114072 | 0.544 | 0.0091602164 | 0.02492310 | -0.07914504 | 0.434 |
| **Max4** |  |  |  |  |  |  |  |  |  |
| Beaver.2:Beaver.3 | 0.802003727 | 36.67813 | 122.93778 | -1.78318224 | 0.969 | 0.001512965 | 0.01929974 | -1.09571150 | 0.879 |
| *Boot.2:Boot.3* | *0.318984164* | *71.39850* | *68.72785* | *1.98059658* | *0.039 ** | *0.065378850* | *0.04254214* | *3.53161962* | *0.005 *** |
| Misty.2:Misty.3 | 0.641259411 | 50.11421 | 112.45548 | -0.99216692 | 0.827 | 0.005156696 | 0.02237824 | -0.56352028 | 0.650 |
| Pye.2:Pye.3 | 0.402745391 | 66.25008 | 89.68910 | 0.62591030 | 0.250 | 0.022424904 | 0.03203185 | 0.94741324 | 0.175 |
| Roberts.2:Roberts.3 | 0.388152473 | 67.16041 | 109.40651 | -0.09446979 | 0.513 | 0.018901429 | 0.03472320 | 0.51182065 | 0.261 |
| VillageBay.2:VillageBay.3 | 0.930154954 | 21.54102 | 38.94957 | -0.29371719 | 0.578 | 0.004753103 | 0.02668685 | -0.78336734 | 0.742 |
| **Total** |  |  |  |  |  |  |  |  |  |
| Beaver.2:Beaver.3 | 0.72814583 | 43.26882 | 116.45220 | -2.18813781 | 0.989 | 0.003071072 | 0.01276396 | -0.57215728 | 0.638 |
| Boot.2:Boot.3 | 0.45547384 | 62.90457 | *61.76713* | *1.91692541* | *0.042 ** | *0.039077269* | *0.03286353* | *2.46613270* | *0.023** |
| Misty.2:Misty.3 | 0.76047425 | 40.49398 | 82.29987 | -1.10867148 | 0.858 | 0.005134991 | 0.01350654 | -0.11484026 | 0.475 |
| Pye.2:Pye.3 | 0.59183954 | 53.71234 | 93.63366 | -0.55121658 | 0.675 | 0.009835958 | 0.02062382 | 0.21307495 | 0.358 |
| Roberts.2:Roberts.3 | 0.52686800 | 58.20592 | 87.85500 | 0.04757294 | 0.442 | 0.017690883 | 0.02694590 | 0.88017043 | 0.171 |
| VillageBay.2:VillageBay.3 | 0.77214474 | 39.45312 | 60.33711 | -0.19699100 | 0.554 | 0.006766905 | 0.01432750 | 0.11830837 | 0.405 |

| **Table S7.** Pairwise comparisons of shape vectors for 2D and 3D datasets of the same watersheds in a common shape space, using diet Simpson diversity as a covariate. Comparisons of angles are on the left, and comparisons of slope vector lengths are on the right. Confidence limits are single-tailed, with null hypotheses for comparisons being 0º, and a distance of 0 units in shape space, respectively. Comparisons were conducted for the opercular four-bar linkage (Op4), the mandibular lever system (Mand), the maxillary four-bar linkage (Max4), and the dataset including all landmarks (Total). Statistically significant comparisons are italicized. Significance codes: (.) 0.1, (*) 0.05, (**) 0.01 | | | | | | | | | |
| --- | --- | --- | --- | --- | --- | --- | --- | --- | --- |
| **Watershed*Simpson*Dim** |  |  |  |  |  |  |  |  |  |
| Comparison | r | angle | UCL (95%) | Z | Pr > angle | d | UCL (95%) | Z | Pr > d |
| **Op4** |  |  |  |  |  |  |  |  |  |
| Beaver.2:Beaver.3 | 0.43874336 | 63.97627 | 125.08282 | -0.40307686 | 0.608 | 1.757575e-01 | 0.3814583 | 0.215181406 | 0.347 |
| *Boot.2:Boot.3* | 0.26689114 | *74.52064* | *50.59488* | *3.88841285* | *0.004 *** | *1.955390e+00* | *1.1122887* | *4.097597424* | *0.003 *** |
| Misty.2:Misty.3 | 0.69169458 | 46.23560 | 57.71828 | 1.03571678 | 0.149 | 3.055394e+00 | 5.9836394 | 0.341582978 | 0.313 |
| Pye.2:Pye.3 | 0.45691621 | 62.81171 | 92.49880 | 0.60226185 | 0.240 | 6.116272e-01 | 0.9703956 | 0.746056291 | 0.206 |
| Roberts.2:Roberts.3 | 0.51955572 | 58.69755 | 75.18920 | 0.98987581 | 0.157 | 4.573956e-01 | 0.7597643 | 0.655652393 | 0.236 |
| VillageBay.2:VillageBay.3 | 0.65164096 | *49.33456* | *46.18616* | *2.26439981* | *0.031 ** | 9.009078e-01 | 1.1482764 | 1.185255710 | 0.131 |
| **Mand** |  |  |  |  |  |  |  |  |  |
| Beaver.2:Beaver.3 | 0.40347446 | 66.20444 | 119.88899 | -0.232654260 | 0.549 | 0.28146838 | 0.5924004 | 0.302720053 | 0.301 |
| Boot.2:Boot.3 | 0.43079314 | 64.48209 | 64.64460 | 1.947560791 | 0.054 . | 1.68757107 | 1.6763034 | 1.976758773 | 0.050 * |
| Misty.2:Misty.3 | 0.77373543 | 39.30948 | 96.78566 | -0.749073420 | 0.757 | 1.79458240 | 8.6782093 | -0.518201323 | 0.623 |
| Pye.2:Pye.3 | 0.46478249 | 62.30385 | 93.18112 | 0.608983247 | 0.235 | 1.05877186 | 1.6711532 | 0.759501850 | 0.198 |
| Roberts.2:Roberts.3 | 0.56785927 | 55.39892 | 117.51546 | -0.406629704 | 0.596 | 0.29011219 | 1.0932964 | -0.330176986 | 0.535 |
| VillageBay.2:VillageBay.3 | 0.61727528 | 51.88257 | 89.54544 | 0.122772536 | 0.388 | 0.74219479 | 1.7091074 | 0.185519378 | 0.340 |
| **Max4** |  |  |  |  |  |  |  |  |  |
| Beaver.2:Beaver.3 | 0.802003727 | 36.67813 | 117.28566 | -1.5108827430 | 0.942 | 0.03538183 | 0.4247558 | -1.03357549 | 0.871 |
| *Boot.2:Boot.3* | 0.318984164 | *71.39850* | *66.25315* | *2.0966700353* | *0.030 ** | *1.96003706* | *1.1680900* | *3.99021781* | *0.001 *** |
| Misty.2:Misty.3 | 0.641259411 | 50.11421 | 102.36548 | -0.5854136768 | 0.685 | 2.12214307 | 9.1544702 | -0.48515814 | 0.613 |
| Pye.2:Pye.3 | 0.402745391 | 66.25008 | 83.44093 | 0.9610787689 | 0.170 | 1.14587258 | 1.4961345 | 1.20789517 | 0.126 |
| Roberts.2:Roberts.3 | 0.388152473 | 67.16041 | 104.84411 | -0.0005720937 | 0.481 | 0.45260129 | 0.8150054 | 0.51957701 | 0.251 |
| VillageBay.2:VillageBay.3 | 0.930154954 | 21.54102 | 33.06755 | 0.2652527378 | 0.344 | 0.38511407 | 2.2101237 | -0.77581663 | 0.748 |
| **Total** |  |  |  |  |  |  |  |  |  |
| Beaver.2:Beaver.3 | 0.72814583 | 43.26882 | 99.76528 | -1.34657227 | 0.920 | 0.07181934 | 0.2581763 | -0.38342570 | 0.569 |
| Boot.2:Boot.3 | 0.45547384 | *62.90457* | *57.16142* | *2.35467360* | *0.019 ** | *1.17152407* | *0.8313119* | *3.19465875* | *0.009 *** |
| Misty.2:Misty.3 | 0.76047425 | 40.49398 | 63.43147 | -0.12207599 | 0.510 | 2.11321074 | 4.3125559 | 0.35983684 | 0.295 |
| Pye.2:Pye.3 | 0.59183954 | 53.71234 | 79.65117 | 0.14896698 | 0.396 | 0.50259988 | 0.8164777 | 0.72673912 | 0.203 |
| Roberts.2:Roberts.3 | 0.52686800 | 58.20592 | 79.02213 | 0.34212572 | 0.334 | 0.42361433 | 0.5419896 | 1.15167344 | 0.143 |
| VillageBay.2:VillageBay.3 | 0.77214474 | 39.45312 | 45.74664 | 1.07286424 | 0.137 | 0.54827981 | 0.8215362 | 0.86667927 | 0.190 |

| **Table S8.** Pairwise comparisons of shape vectors for 2D and 3D datasets across all watersheds in a common shape space, using diet type as a covariate. Comparisons of angles are on the left, and comparisons of vector magnitudes are on the right. Confidence limits are single-tailed, with null hypotheses for comparisons being 0º, and a distance of 0 units in shape space, respectively. Comparisons were conducted for the opercular four-bar linkage (Op4), the mandibular lever system (Mand), the maxillary four-bar linkage (Max4), and the dataset including all landmarks (Total). Statistically significant comparisons are italicized. Significance codes: (.) 0.1, (*) 0.05, (**) 0.01 | | | | | | | | | |
| --- | --- | --- | --- | --- | --- | --- | --- | --- | --- |
| **LD*Dim** | r | angle | UCL (95%) | Z | Pr > angle | d | UCL (95%) | Z | Pr > d |
| Op4 | *0.4564667* | *62.84066* | *42.29231* | *3.900653* | *0.002 *** | *0.01410849* | *0.01028839* | *3.066788* | *0.007 *** |
| Mand | 0.4888028 | 60.73808 | 66.97127 | 1.569116 | 0.082 . | 0.01172413 | 0.01425147 | 1.367796 | 0.107 |
| Max4 | 0.6249066 | 51.32467 | 68.58997 | 0.7873927 | 0.199 | 0.006243088 | 0.01408703 | 0.06860461 | 0.413 |
| Total | *0.581044* | *54.476* | *45.53927* | *2.861319* | *0.009 *** | *0.009191175* | *0.007425044* | *2.680486* | *0.013 ** |

| **Table S9.** Pairwise comparisons of shape vectors for 2D and 3D datasets across all watersheds in a common shape space, using diet Simpson diversity as a covariate. Comparisons of angles are on the left, and comparisons of vector magnitudes are on the right. Confidence limits are single-tailed, with null hypotheses for comparisons being 0º, and a distance of 0 units in shape space, respectively. Comparisons were conducted for the opercular four-bar linkage (Op4), the mandibular lever system (Mand), the maxillary four-bar linkage (Max4), and the dataset including all landmarks (Total). Statistically significant comparisons are italicized. Significance codes: (.) 0.1, (*) 0.05, (**) 0.01 | | | | | | | | | |
| --- | --- | --- | --- | --- | --- | --- | --- | --- | --- |
| **Simpson*Dim** | r | angle | UCL (95%) | Z | Pr > angle | d | UCL (95%) | Z | Pr > d |
| Op4 | 0.2395684 | 76.13893 | 86.79457 | 1.419499 | 0.102 | *0.298527* | *0.2508351* | *2.469068* | *0.025 ** |
| Mand | 0.2350317 | 76.40651 | 118.9649 | 0.2615082 | 0.385 | 0.1930926 | 0.2477843 | 1.204321 | 0.121 |
| Max4 | 0.751868 | 41.24755 | 101.3249 | -0.7979668 | 0.754 | 0.03392524 | 0.2636788 | -0.9285473 | 0.813 |
| Total | 0.7035989 | 45.28354 | 63.59082 | 0.2954441 | 0.346 | 0.0883845 | 0.1677574 | 0.3821863 | 0.291 |

| **Table S10.** ANCOVAs of opercular four-bar linkage KT residuals of total centroid size. All ANCOVAs include watershed as a factor and diet type (left) or Gini-Simpson diversity (right) as covariates. Identical ANCOVA models were performed on both 2D and 3D landmark datasets (first three rows and last three rows, respectively). KT calculations were made from kinematic models using planar kinematics (rows 1 and 4), with the rotational axis of the input joint oriented anteriorly 15º (rows 2 and 5), and with the rotational axis oriented anteriorly 30º (rows 3 and 6). Effects use type II sums of squares. Significance codes: (.) 0.1, (*) 0.05, (**) 0.01, (***) 0.001 | | | | | | | | | | | | | | |
| --- | --- | --- | --- | --- | --- | --- | --- | --- | --- | --- | --- | --- | --- | --- |
| **Op4 KT** | | | | | | | | | | | | | | |
| Op4.2d.KT.res | Sum Sq | Df | F value | Pr(>F) | Op4.2d.KT.res | Sum Sq | Df | F value | Pr(>F) | Op4.2d.KT.res | Sum Sq | Df | F value | Pr(>F) |
| Watershed | 0.125119 | 5 | 10.1591 | 1.219e-06 *** | Watershed | 0.115783 | 5 | 9.4011 | 2.929e-06 *** | Watershed | 0.100123 | 5 | 8.1296 | 1.369e-05 *** |
| LD1 | 0.013069 | 1 | 5.3058 | 0.0257247 * | Habitat | 0.017578 | 1 | 7.1364 | 0.0103456 * | simpson | 0.004894 | 1 | 1.9867 | 0.1652670 |
| Watershed:LD1 | 0.071385 | 5 | 5.7962 | 0.0003012 *** | Watershed:Habitat | 0.066876 | 5 | 5.4301 | 0.0005057 *** | Watershed:simpson | 0.079561 | 5 | 6.4600 | 0.0001205 *** |
| Residuals | 0.115770 | 47 |  |  | Residuals | 0.115770 | 47 |  |  | Residuals | 0.115770 | 47 |  |  |
| Op4.2d.KT.15.res |  |  |  |  | Op4.2d.KT.15.res |  |  |  |  | Op4.2d.KT.15.res |  |  |  |  |
| Watershed | 0.124908 | 5 | 10.1758 | 1.196e-06 *** | Watershed | 0.115331 | 5 | 9.3956 | 2.948e-06 *** | Watershed | 0.099735 | 5 | 8.1251 | 1.377e-05 *** |
| LD1 | 0.013563 | 1 | 5.5245 | 0.023001 * | Habitat | 0.018137 | 1 | 7.3876 | 0.0091682 ** | simpson | 0.004796 | 1 | 1.9534 | 0.1687854 |
| Watershed:LD1 | 0.071389 | 5 | 5.8158 | 0.000293 *** | Watershed:Habitat | 0.066815 | 5 | 5.4431 | 0.0004964 *** | Watershed:simpson | 0.080156 | 5 | 6.5300 | 0.0001096 *** |
| Residuals | 0.115385 | 47 |  |  | Residuals | 0.115385 | 47 |  |  | Residuals | 0.115385 | 47 |  |  |
| Op4.2d.KT.30.res |  |  |  |  | Op4.2d.KT.30.res |  |  |  |  | Op4.2d.KT.30.res |  |  |  |  |
| Watershed | 0.124199 | 5 | 10.2036 | 1.159e-06 *** | Watershed | 0.113978 | 5 | 9.3639 | 3.06e-06 *** | Watershed | 0.098497 | 5 | 8.0921 | 1.434e-05 *** |
| LD1 | 0.014958 | 1 | 6.1443 | 0.0168292 * | Habitat | 0.019702 | 1 | 8.0930 | 0.0065618 ** | simpson | 0.004559 | 1 | 1.8729 | 0.1777 |
| Watershed:LD1 | 0.071543 | 5 | 5.8776 | 0.0002687 *** | Watershed:Habitat | 0.066799 | 5 | 5.4879 | 0.0004657 *** | Watershed:simpson | 0.081941 | 5 | 6.7319 | 8.352e-05 *** |
| Residuals | 0.114417 | 47 |  |  | Residuals | 0.114417 | 47 |  |  | Residuals | 0.114417 | 47 |  |  |
| Op4.3d.KT.res |  |  |  |  | Op4.3d.KT.res |  |  |  |  | Op4.3d.KT.res |  |  |  |  |
| Watershed | 0.124470 | 5 | 9.4545 | 2.75e-06 *** | Watershed | 0.113388 | 5 | 8.6127 | 7.535e-06 *** | Watershed | 0.098668 | 5 | 7.4946 | 3.065e-05 *** |
| LD1 | 0.016303 | 1 | 6.1916 | 0.0164374 * | Habitat | 0.021872 | 1 | 8.3066 | 0.005938 ** | simpson | 0.004952 | 1 | 1.8808 | 0.176752 |
| Watershed:LD1 | 0.075039 | 5 | 5.6998 | 0.0003449 *** | Watershed:Habitat | 0.069470 | 5 | 5.2768 | 0.000630 *** | Watershed:simpson | 0.086390 | 5 | 6.5620 | 0.000105 *** |
| Residuals | 0.123753 | 47 |  |  | Residuals | 0.123753 | 47 |  |  | Residuals | 0.123753 | 47 |  |  |
| Op4.3d.KT.15.res |  |  |  |  | Op4.3d.KT.15.res |  |  |  |  | Op4.3d.KT.15.res |  |  |  |  |
| Watershed | 0.125108 | 5 | 9.7360 | 1.981e-06 *** | Watershed | 0.115774 | 5 | 9.0097 | 4.661e-06 *** | Watershed | 0.100705 | 5 | 7.8369 | 1.979e-05 *** |
| LD1 | 0.012890 | 1 | 5.0154 | 0.0298933 * | Habitat | 0.017539 | 1 | 6.8245 | 0.0120354 * | simpson | 0.004155 | 1 | 1.6166 | 0.2098261 |
| Watershed:LD1 | 0.069963 | 5 | 5.4445 | 0.0004954 *** | Watershed:Habitat | 0.065313 | 5 | 5.0827 | 0.0008341 *** | Watershed:simpson | 0.078698 | 5 | 6.1243 | 0.0001908 *** |
| Residuals | 0.120790 | 47 |  |  | Residuals | 0.120790 | 47 |  |  | Residuals | 0.120790 | 47 |  |  |
| Op4.3d.KT.30.res |  |  |  |  | Op4.3d.KT.30.res |  |  |  |  | Op4.3d.KT.30.res |  |  |  |  |
| Watershed | 0.125571 | 5 | 10.0755 | 1.341e-06 *** | Watershed | 0.117770 | 5 | 9.4496 | 2.766e-06 *** | Watershed | 0.102080 | 5 | 8.1906 | 1.268e-05 *** |
| LD1 | 0.010306 | 1 | 4.1347 | 0.0476808 * | Habitat | 0.014070 | 1 | 5.6446 | 0.0216378 * | simpson | 0.003509 | 1 | 1.4078 | 0.2413857 |
| Watershed:LD1 | 0.066278 | 5 | 5.3179 | 0.0005938 *** | Watershed:Habitat | 0.062514 | 5 | 5.0160 | 0.0009191 *** | Watershed:simpson | 0.073075 | 5 | 5.8633 | 0.0002742 *** |
| Residuals | 0.117152 | 47 |  |  | Residuals | 0.117152 | 47 |  |  | Residuals | 0.117152 | 47 |  |  |

| **Table S11.** ANCOVAs of maxillary four-bar linkage KT residuals of total centroid size. All ANCOVAs include watershed as a factor and diet type (left) or Gini-Simpson diversity (right) as covariates. Identical ANCOVA models were performed on both 2D and 3D landmark datasets (first three rows and last three rows, respectively). KT calculations were made from kinematic models using planar kinematics (rows 1 and 4), with the rotational axis of the input joint oriented posteriorly 10º (rows 2 and 5), and with the rotational axis oriented anteriorly 20º (rows 3 and 6). Effects use type II sums of squares. Significance codes: (.) 0.1, (*) 0.05, (**) 0.01, (***) 0.001 | | | | | | | | | | | | | | |
| --- | --- | --- | --- | --- | --- | --- | --- | --- | --- | --- | --- | --- | --- | --- |
| **Max4** | | | | | | | | | | | | | | |
| Max4.2d.KT.res | Sum Sq | Df | F value | Pr(>F) | Max4.2d.KT.res | Sum Sq | Df | F value | Pr(>F) | Max4.2d.KT.res | Sum Sq | Df | F value | Pr(>F) |
| Watershed | 0.114246 | 5 | 8.0002 | 1.610e-05 *** | Watershed | 0.112125 | 5 | 7.8517 | 1.942e-05 *** | Watershed | 0.077500 | 5 | 5.4270 | 0.0005079 *** |
| LD1 | 0.002668 | 1 | 0.9340 | 0.3388 | Habitat | 0.003304 | 1 | 1.1568 | 0.2876 | simpson | 0.024087 | 1 | 8.4336 | 0.0055965 ** |
| Watershed:LD1 | 0.135397 | 5 | 9.4813 | 2.665e-06 *** | Watershed:Habitat | 0.134760 | 5 | 9.4368 | 2.808e-06 *** | Watershed:simpson | 0.113977 | 5 | 7.9814 | 1.648e-05 *** |
| Residuals | 0.134235 | 47 |  |  | Residuals | 0.134235 | 47 |  |  | Residuals | 0.134235 | 47 |  |  |
| Max4.2d.KT.10.res |  |  |  |  | Max4.2d.KT.10.res |  |  |  |  | Max4.2d.KT.10.res |  |  |  |  |
| Watershed | 0.114253 | 5 | 8.0003 | 1.610e-05 *** | Watershed | 0.112132 | 5 | 7.8517 | 1.942e-05 *** | Watershed | 0.077508 | 5 | 5.4273 | 0.0005077 *** |
| LD1 | 0.002668 | 1 | 0.9341 | 0.3387 | Habitat | 0.003305 | 1 | 1.1573 | 0.2875 | simpson | 0.024087 | 1 | 8.4331 | 0.0055977 ** |
| Watershed:LD1 | 0.135401 | 5 | 9.4811 | 2.666e-06 *** | Watershed:Habitat | 0.134764 | 5 | 9.4365 | 2.809e-06 *** | Watershed:simpson | 0.113982 | 5 | 7.9813 | 1.649e-05 *** |
| Residuals | 0.134243 | 47 |  |  | Residuals | 0.134243 | 47 |  |  | Residuals | 0.134243 | 47 |  |  |
| Max4.2d.KT.20.res |  |  |  |  | Max4.2d.KT.20.res |  |  |  |  | Max4.2d.KT.20.res |  |  |  |  |
| Watershed | 0.11428 | 5 | 8.0006 | 1.609e-05 *** | Watershed | 0.11216 | 5 | 7.8520 | 1.941e-05 *** | Watershed | 0.077535 | 5 | 5.4282 | 0.0005071 *** |
| LD1 | 0.00267 | 1 | 0.9345 | 0.3386 | Habitat | 0.00331 | 1 | 1.1588 | 0.2872 | simpson | 0.024087 | 1 | 8.4316 | 0.0056017 ** |
| Watershed:LD1 | 0.13542 | 5 | 9.4804 | 2.668e-06 *** | Watershed:Habitat | 0.13477 | 5 | 9.4356 | 2.812e-06 *** | Watershed:simpson | 0.113998 | 5 | 7.9810 | 1.649e-05 *** |
| Residuals | 0.13427 | 47 |  |  | Residuals | 0.13427 | 47 |  |  | Residuals | 0.134266 | 47 |  |  |
| Max4.3d.KT.res |  |  |  |  | Max4.3d.KT.res |  |  |  |  | Max4.3d.KT.res |  |  |  |  |
| Watershed | 0.114499 | 5 | 7.3297 | 3.795e-05 *** | Watershed | 0.110486 | 5 | 7.0728 | 5.311e-05 *** | Watershed | 0.078976 | 5 | 5.0557 | 0.0008675 *** |
| LD1 | 0.005043 | 1 | 1.6142 | 0.2102 | Habitat | 0.006693 | 1 | 2.1422 | 0.15 | simpson | 0.022813 | 1 | 7.3019 | 0.0095529 ** |
| Watershed:LD1 | 0.137663 | 5 | 8.8125 | 5.910e-06 *** | Watershed:Habitat | 0.136013 | 5 | 8.7069 | 6.718e-06 *** | Watershed:simpson | 0.119893 | 5 | 7.6750 | 2.432e-05 *** |
| Residuals | 0.146840 | 47 |  |  | Residuals | 0.146840 | 47 |  |  | Residuals | 0.146840 | 47 |  |  |
| Max4.3d.KT.10.res |  |  |  |  | Max4.3d.KT.10.res |  |  |  |  | Max4.3d.KT.10.res |  |  |  |  |
| Watershed | 0.113733 | 5 | 7.3504 | 3.694e-05 *** | Watershed | 0.11000 | 5 | 7.1090 | 5.064e-05 *** | Watershed | 0.077777 | 5 | 5.0266 | 0.000905 *** |
| LD1 | 0.004714 | 1 | 1.5234 | 0.2232 | Habitat | 0.00610 | 1 | 1.9712 | 0.1669 | simpson | 0.023421 | 1 | 7.5684 | 0.008409 ** |
| Watershed:LD1 | 0.138097 | 5 | 8.9250 | 5.160e-06 *** | Watershed:Habitat | 0.13671 | 5 | 8.8355 | 5.748e-06 *** | Watershed:simpson | 0.119390 | 5 | 7.7160 | 2.307e-05 *** |
| Residuals | 0.145446 | 47 |  |  | Residuals | 0.14545 | 47 |  |  | Residuals | 0.145446 | 47 |  |  |
| Max4.3d.KT.20.res |  |  |  |  | Max4.3d.KT.20.res |  |  |  |  | Max4.3d.KT.20.res |  |  |  |  |
| Watershed | 0.114454 | 5 | 7.3395 | 3.747e-05 *** | Watershed | 0.110479 | 5 | 7.0846 | 5.229e-05 *** | Watershed | 0.078791 | 5 | 5.0526 | 0.0008714 *** |
| LD1 | 0.005003 | 1 | 1.6041 | 0.2116 | Habitat | 0.006617 | 1 | 2.1216 | 0.1519 | simpson | 0.022910 | 1 | 7.3456 | 0.0093546 ** |
| Watershed:LD1 | 0.137818 | 5 | 8.8378 | 5.732e-06 *** | Watershed:Habitat | 0.136204 | 5 | 8.7343 | 6.498e-06 *** | Watershed:simpson | 0.119911 | 5 | 7.6895 | 2.387e-05 *** |
| Residuals | 0.146585 | 47 |  |  | Residuals | 0.146585 | 47 |  |  | Residuals | 0.146585 | 47 |  |  |

| **Table S12.** ANCOVAs of opening (first two rows) and closing (last two rows) LR residuals of total centroid size. All ANCOVAs include watershed as a factor and diet type (left) or Gini-Simpson diversity (right) as covariates. Identical ANCOVA models were performed on both 2D and 3D landmark datasets (rows 1 and 3 and rows 2 and 4, respectively). Effects use type II sums of squares. Significance codes: (.) 0.1, (*) 0.05, (**) 0.01, (***) 0.001 | | | | | | | | | | | | | | |
| --- | --- | --- | --- | --- | --- | --- | --- | --- | --- | --- | --- | --- | --- | --- |
| **Mand** | | | | | | | | | | | | | | |
| Mand 2D Opening | Sum Sq | Df | F value | Pr(>F) | Mand 2D Opening | Sum Sq | Df | F value | Pr(>F) | Mand 2D Opening | Sum Sq | Df | F value | Pr(>F) |
| Watershed | *0.124157* | *5* | *8.8090* | *5.936e-06 **** | Watershed | 0.119760 | 5 | 8.4970 | 8.682e-06 *** | Watershed | *0.083984* | *5* | *5.9587* | *0.000240 **** |
| LD1 | 0.005558 | 1 | 1.9718 | 0.1668 | Habitat | 0.007336 | 1 | 2.6025 | 0.1134 | simpson | *0.021885* | *1* | *7.7636* | *0.007665 *** |
| Watershed:LD1 | *0.130003* | *5* | *9.2238* | *3.611e-06 **** | Watershed:Habitat | 0.128225 | 5 | 9.0976 | 4.196e-06 *** | Watershed:simpson | *0.113677* | *5* | *8.0654* | *1.483e-05 **** |
| Residuals | 0.132487 | 47 |  |  | Residuals | 0.132487 | 47 |  |  | Residuals | 0.132487 | 47 |  |  |
| Mand 3D Opening |  |  |  |  | Mand 3D Opening |  |  |  |  |  |  |  |  |  |
| Watershed | *0.120082* | *5* | *7.8549* | *1.934e-05 **** | Watershed | 0.114762 | 5 | 7.5069 | 3.017e-05 *** | Watershed | *0.081808* | *5* | *5.3513* | *0.0005661 **** |
| LD1 | 0.006925 | 1 | 2.2650 | 0.139 | Habitat | 0.009172 | 1 | 2.9999 | 0.08983 . | simpson | *0.021911* | *1* | *7.1664* | *0.0101967 ** |
| Watershed:LD1 | *0.134051* | *5* | *8.7686* | *6.233e-06 **** | Watershed:Habitat | 0.131804 | 5 | 8.6216 | 7.453e-06 *** | Watershed:simpson | *0.119065* | *5* | *7.7883* | *2.105e-05 **** |
| Residuals | 0.143703 | 47 |  |  | Residuals | 0.143703 | 47 |  |  | Residuals | 0.143703 | 47 |  |  |
| Mand 2D Closing |  |  |  |  | Mand 2D Closing |  |  |  |  |  |  |  |  |  |
| Watershed | *0.097156* | *5* | *7.1480* | *4.811e-05 **** | Watershed | 0.097670 | 5 | 7.1858 | 4.579e-05 *** | Watershed | *0.062028* | *5* | *4.5636* | *0.001789 *** |
| LD1 | 0.000437 | 1 | 0.1606 | 0.6904 | Habitat | 0.000349 | 1 | 0.1285 | 0.7216 | simpson | *0.023278* | *1* | *8.5632* | *0.005270 *** |
| Watershed:LD1 | *0.128243* | *5* | *9.4352* | *2.814e-06 **** | Watershed:Habitat | 0.128331 | 5 | 9.4416 | 2.792e-06 *** | Watershed:simpson | *0.105402* | *5* | *7.7546* | *2.197e-05 **** |
| Residuals | 0.127765 | 47 |  |  | Residuals | 0.127765 | 47 |  |  | Residuals | 0.127765 | 47 |  |  |
| Mand 3D Closing |  |  |  |  | Mand 3D Closing |  |  |  |  |  |  |  |  |  |
| Watershed | *0.093938* | *5* | *6.4727* | *0.0001184 **** | Watershed | 0.093361 | 5 | 6.4330 | 0.000125 *** | Watershed | *0.059536* | *5* | *4.1023* | *0.00358 *** |
| LD1 | 0.001098 | 1 | 0.3783 | 0.5414834 | Habitat | 0.001049 | 1 | 0.3614 | 0.550627 | simpson | *0.023091* | *1* | *7.9555* | *0.00700 *** |
| Watershed:LD1 | *0.129991* | *5* | *8.9569* | *4.966e-06 **** | Watershed:Habitat | 0.130040 | 5 | 8.9603 | 4.946e-06 *** | Watershed:simpson | *0.107997* | *5* | *7.4415* | *3.283e-05 **** |
| Residuals | 0.136421 | 47 |  |  | Residuals | 0.136421 | 47 |  |  | Residuals | 0.136421 | 47 |  |  |

| **Table S13.** PERMANOVAs including centroid size residuals of all biomechanical estimates as dependent variables. All PERMANOVAs include watershed as a factor and diet type (left) or Gini-Simpson diversity (right) as covariates. Identical models were performed on both 2D and 3D landmark datasets (rows 1-3 and rows 4-6, respectively). Effects use type II sums of squares. Kinematic model names include the orientation of rotational angles in the Op4 and Max4 kinematic (Op4º/Max4º) models. Significance codes: (.) 0.1, (*) 0.05, (**) 0.01, (***) 0.001. | | | | | | | | | | | | | | | |
| --- | --- | --- | --- | --- | --- | --- | --- | --- | --- | --- | --- | --- | --- | --- | --- |
| **KT PERMANOVAs** | | | | | | | | | | | | | | | |
| **Diet LD models** | | | | | | | | **Gini-Simpson diversity models** | | | | | | | |
| 2D 0º/0º | **Df** | **SS** | **MS** | **Rsq** | **F** | **Z** | **Pr(>F)** |  | **Df** | **SS** | **MS** | **Rsq** | **F** | **Z** | **Pr(>F)** |
| Watershed | *5* | *0.46068* | *0.092135* | *0.32003* | *8.4866* | *3.3355* | *0.001 *** | Watershed | *5* | *0.32364* | *0.064727* | *0.22483* | *5.9620* | *2.8651* | *0.001 *** |
| LD1 | 1 | 0.02173 | 0.021732 | 0.01510 | 2.0017 | 0.9935 | 0.169 | simpson | *1* | *0.07414* | *0.074143* | *0.05151* | *6.8294* | *1.9265* | *0.011 ** |
| Watershed:LD1 | *5* | *0.46503* | *0.093006* | *0.32305* | *8.5668* | *3.3716* | *0.001 *** | Watershed:simpson | *5* | *0.41262* | *0.082523* | *0.28664* | *7.6012* | *3.3046* | *0.001 *** |
| Residuals | 47 | 0.51026 | 0.010857 | 0.35447 |  |  |  | Residuals | 47 | 0.51026 | 0.010857 | 0.35447 |  |  |  |
| Total | 58 | 1.43948 |  |  |  |  |  | Total | 58 | 1.43948 |  |  |  |  |  |
| 2D 15º/10º |  |  |  |  |  |  |  |  |  |  |  |  |  |  |  |
| Watershed | *5* | *0.46047* | *0.092095* | *0.31997* | *8.4892* | *3.3360* | *0.001 *** | Watershed | *5* | *0.32326* | *0.064651* | *0.22462* | *5.9594* | *2.8647* | *0.001 *** |
| LD1 | 1 | 0.02223 | 0.022226 | 0.01544 | 2.0487 | 1.0088 | 0.163 | simpson | *1* | *0.07405* | *0.074045* | *0.05145* | *6.8254* | *1.9261* | *0.011 ** |
| Watershed:LD1 | *5* | *0.46504* | *0.093007* | *0.32314* | *8.5733* | *3.3721* | *0.001 *** | Watershed:simpson | *5* | *0.41322* | *0.082643* | *0.28713* | *7.6179* | *3.3085* | *0.001 *** |
| Residuals | 47 | 0.50988 | 0.010849 | 0.35430 |  |  |  | Residuals | 47 | 0.50988 | 0.010849 | 0.35430 |  |  |  |
| Total | 58 | 1.43914 |  |  |  |  |  | Total | 58 | 1.43914 |  |  |  |  |  |
| 2D 30º/20º |  |  |  |  |  |  |  |  |  |  |  |  |  |  |  |
| Watershed | *5* | *0.45979* | *0.091958* | *0.31966* | *8.4923* | *3.3364* | *0.001 *** | Watershed | *5* | *0.32204* | *0.064409* | *0.22390* | *5.9481* | *2.8624* | *0.001 *** |
| LD1 | 1 | 0.02362 | 0.023622 | 0.01642 | 2.1815 | 1.0503 | 0.152 | simpson | *1* | *0.07381* | *0.073809* | *0.05131* | *6.8162* | *1.9247* | *0.011 ** |
| Watershed:LD1 | *5* | *0.46520* | *0.093041* | *0.32343* | *8.5923* | *3.3737* | *0.001 *** | Watershed:simpson | *5* | *0.41502* | *0.083003* | *0.28853* | *7.6653* | *3.3198* | *0.001 *** |
| Residuals | 47 | 0.50894 | 0.010828 | 0.35383 |  |  |  | Residuals | 47 | 0.50894 | 0.010828 | 0.35383 |  |  |  |
| Total | 58 | 1.43836 |  |  |  |  |  | Total | 58 | 1.43836 |  |  |  |  |  |
| 3D 0º/0º |  |  |  |  |  |  |  |  |  |  |  |  |  |  |  |
| Watershed | *5* | *0.45299* | *0.090598* | *0.30493* | *7.7319* | *3.2158* | *0.001 *** | Watershed | *5* | *0.31899* | *0.063798* | *0.21473* | *5.4447* | *2.7429* | *0.001 *** |
| LD1 | 1 | 0.02937 | 0.029369 | 0.01977 | 2.5064 | 1.1386 | 0.124 | simpson | *1* | *0.07277* | *0.072768* | *0.04898* | *6.2103* | *1.8545* | *0.014 ** |
| Watershed:LD1 | *5* | *0.47674* | *0.095349* | *0.32092* | *8.1374* | *3.3107* | *0.001 *** | Watershed:simpson | *5* | *0.43334* | *0.086669* | *0.29170* | *7.3966* | *3.2841* | *0.001 *** |
| Residuals | 47 | 0.55072 | 0.011717 | 0.37071 |  |  |  | Residuals | 47 | 0.55072 | 0.011717 | 0.37071 |  |  |  |
| Total | 58 | 1.48556 |  |  |  |  |  | Total | 58 | 1.48556 |  |  |  |  |  |
| 3D 15º/10º |  |  |  |  |  |  |  |  |  |  |  |  |  |  |  |
| Watershed | *5* | *0.45286* | *0.090572* | *0.30706* | *7.7914* | *3.2383* | *0.001 *** | Watershed | *5* | *0.31983* | *0.063965* | *0.21685* | *5.5025* | *2.7726* | *0.001 *** |
| LD1 | 1 | 0.02563 | 0.025627 | 0.01738 | 2.2046 | 1.0568 | 0.152 | simpson | *1* | *0.07258* | *0.072579* | *0.04921* | *6.2435* | *1.8682* | *0.014 ** |
| Watershed:LD1 | *5* | *0.47210* | *0.094420* | *0.32010* | *8.1224* | *3.3268* | *0.001 *** | Watershed:simpson | *5* | *0.42515* | *0.085030* | *0.28827* | *7.3146* | *3.2734* | *0.001 *** |
| Residuals | 47 | 0.54636 | 0.011625 | 0.37045 |  |  |  | Residuals | 47 | 0.54636 | 0.011625 | 0.37045 |  |  |  |
| Total | 58 | 1.47485 |  |  |  |  |  | Total | 58 | 1.47485 |  |  |  |  |  |
| 3D 30º/20º |  |  |  |  |  |  |  |  |  |  |  |  |  |  |  |
| Watershed | *5* | *0.45404* | *0.090809* | *0.30916* | *7.8476* | *3.2690* | *0.001 *** | Watershed | *5* | *0.32222* | *0.064443* | *0.21939* | *5.5691* | *2.8095* | *0.001 *** |
| LD1 | 1 | 0.02333 | 0.023332 | 0.01589 | 2.0164 | 0.9993 | 0.169 | simpson | *1* | *0.07142* | *0.071422* | *0.04863* | *6.1722* | *1.8668* | *0.014 ** |
| Watershed:LD1 | *5* | *0.46814* | *0.093627* | *0.31875* | *8.0912* | *3.3485* | *0.001 *** | Watershed:simpson | *5* | *0.42005* | *0.084009* | *0.28601* | *7.2600* | *3.2793* | *0.001 *** |
| Residuals | 47 | 0.54386 | 0.011572 | 0.37031 |  |  |  | Residuals | 47 | 0.54386 | 0.011572 | 0.37031 |  |  |  |
| Total | 58 | 1.46865 |  |  |  |  |  | Total | 58 | 1.46865 |  |  |  |  |  |

| **Table S14.** ANCOVAs including disparities between estimates from 3D and 2D landmark data of all biomechanical estimate residuals as dependent variables. All ANCOVAs include watershed as a factor and diet type (left) or Gini-Simpson diversity (right) as covariates. Model names indicate lever system and rotational axis orientations (no number between “KT” and “res” indicates planar kinematics; 3d*X*_2d indicates disparity between model from 3D landmarks with 3D kinematics and model from 2D landmarks with planar kinematics). Effects use type II sums of squares. Significance codes: (.) 0.1, (*) 0.05, (**) 0.01, (***) 0.001. | | | | | | | | | |
| --- | --- | --- | --- | --- | --- | --- | --- | --- | --- |
| **Diet LD Score Biomechanical Disparity Models** | | | | | **Simpson Diversity Biomechanical Disparity Models** | | | | |
| **Op4.KT.res.disp** | **Sum Sq** | **Df** | **F value** | **Pr(>F)** | **Op4.KT.res.disp** | **Sum Sq** | **Df** | **F value** | **Pr(>F)** |
| Watershed | *0.00035193* | *5* | *5.2001* | *0.0007037 **** | Watershed | *0.00032780* | *5* | *4.8435* | *0.001183 *** |
| LD1 | *0.00017852* | *1* | *13.1889* | *0.0006943 **** | simpson | 0.00000017 | 1 | 0.0129 | 0.910053 |
| Watershed:LD1 | 0.00011275 | 5 | 1.6659 | 0.1614289 | Watershed:simpson | *0.00029109* | *5* | *4.3011* | *0.002650 *** |
| Residuals | 0.00063617 | 47 |  |  | Residuals | 0.00063617 | 47 |  |  |
| **Op4.KT.15.res.disp** |  |  |  |  | **Op4.KT.15.res.disp** |  |  |  |  |
| Watershed | 3.294e-05 | 5 | 0.6263 | 0.6804 | Watershed | 4.478e-05 | 5 | 0.8513 | 0.5207 |
| LD1 | 8.570e-06 | 1 | 0.8142 | 0.3715 | simpson | 2.298e-05 | 1 | 2.1846 | 0.1461 |
| Watershed:LD1 | 8.771e-05 | 5 | 1.6676 | 0.1610 | Watershed:simpson | 7.329e-05 | 5 | 1.3935 | 0.2440 |
| Residuals | 4.944e-04 | 47 |  |  | Residuals | 4.944e-04 | 47 |  |  |
| **Op4.KT.30.res.disp** |  |  |  |  | **Op4.KT.30.res.disp** |  |  |  |  |
| Watershed | *0.00059402* | *5* | *3.3569* | *0.01128 ** | Watershed | *0.00069001* | *5* | *3.8994* | *0.004877 *** |
| LD1 | *0.00043192* | *1* | *12.2043* | *0.00105 *** | simpson | 0.00006865 | 1 | 1.9399 | 0.170237 |
| Watershed:LD1 | 0.00037626 | 5 | 2.1264 | 0.07866 . | Watershed:simpson | *0.00073953* | *5* | *4.1792* | *0.003186 *** |
| Residuals | 0.00166336 | 47 |  |  | Residuals | 0.00166336 | 47 |  |  |
| **Op4.KT.3d15_2d.res.disp** |  |  |  |  | **Op4.KT.3d15_2d.res.disp** |  |  |  |  |
| Watershed | 0.00002883 | 5 | 0.6779 | 0.64228 | Watershed | 0.00003809 | 5 | 0.8958 | 0.49172 |
| LD1 | 0.00000062 | 1 | 0.0732 | 0.78798 | simpson | 0.00003023 | 1 | 3.5552 | 0.06555 . |
| Watershed:LD1 | 0.00009346 | 5 | 2.1981 | 0.07024 . | Watershed:simpson | 0.00006385 | 5 | 1.5017 | 0.20740 |
| Residuals | 0.00039968 | 47 |  |  | Residuals | 0.00039968 | 47 |  |  |
| **Op4.KT.3d30_2d.res.disp** |  |  |  |  | **Op4.KT.3d30_2d.res.disp** |  |  |  |  |
| Watershed | *0.00048883* | *5* | *3.7310* | *0.006317 *** | Watershed | *0.00058652* | *5* | *4.4767* | *0.002037 *** |
| LD1 | *0.00016388* | *1* | *6.2543* | *0.015933 ** | simpson | *0.00011486* | *1* | *4.3834* | *0.041712 ** |
| Watershed:LD1 | *0.00037594* | *5* | *2.8694* | *0.024269 ** | Watershed:simpson | *0.00042496* | *5* | *3.2436* | *0.013472 ** |
| Residuals | 0.00123156 | 47 |  |  | Residuals | 0.00123156 | 47 |  |  |
| **Max4.KT.res.disp** |  |  |  |  | **Max4.KT.res.disp** |  |  |  |  |
| Watershed | 0.00050928 | 5 | 1.6654 | 0.16156 | Watershed | 0.00030963 | 5 | 1.0126 | 0.42097 |
| LD1 | *0.00037503* | *1* | *6.1320* | *0.01693 ** | simpson | 0.00001730 | 1 | 0.2829 | 0.59732 |
| Watershed:LD1 | 0.00051906 | 5 | 1.6974 | 0.15379 | Watershed:simpson | *0.00087679* | *5* | *2.8672* | *0.02435 ** |
| Residuals | 0.00287448 | 47 |  |  | Residuals | 0.00287448 | 47 |  |  |
| **Max4.KT.10.res.disp** |  |  |  |  | **Max4.KT.10.res.disp** |  |  |  |  |
| Watershed | *0.00038474* | *5* | *3.4442* | *0.0098487 *** | Watershed | *0.00028157* | *5* | *2.5207* | *0.0421470 ** |
| LD1 | *0.00028930* | *1* | *12.9493* | *0.0007672 **** | simpson | 0.00000466 | 1 | 0.2087 | 0.6498942 |
| Watershed:LD1 | *0.00027726* | *5* | *2.4821* | *0.0448050 ** | Watershed:simpson | *0.00056189* | *5* | *5.0302* | *0.0009003 **** |
| Residuals | 0.00105002 | 47 |  |  | Residuals | 0.00105002 | 47 |  |  |
| **Max4.KT.20.res.disp** |  |  |  |  | **Max4.KT.20.res.disp** |  |  |  |  |
| Watershed | 0.00047007 | 5 | 1.7763 | 0.13612 | Watershed | 0.00029030 | 5 | 1.0970 | 0.37468 |
| LD1 | *0.00036341* | *1* | *6.8662* | *0.01179 ** | simpson | 0.00001474 | 1 | 0.2784 | 0.60021 |
| Watershed:LD1 | 0.00047300 | 5 | 1.7874 | 0.13380 | Watershed:simpson | *0.00082166* | *5* | *3.1049* | *0.01674 ** |
| Residuals | 0.00248754 | 47 |  |  | Residuals | 0.00248754 | 47 |  |  |
| **Max4.KT.3d10_2d.res.disp** |  |  |  |  | **Max4.KT.3d10_2d.res.disp** |  |  |  |  |
| Watershed | *0.00038452* | *5* | *3.4242* | *0.0101610 ** | Watershed | *0.00028122* | *5* | *2.5042* | *0.0432582 ** |
| LD1 | *0.00028945* | *1* | *12.8879* | *0.0007872 **** | simpson | 0.00000466 | 1 | 0.2076 | 0.6507449 |
| Watershed:LD1 | *0.00027830* | *5* | *2.4783* | *0.0450752 ** | Watershed:simpson | *0.00056309* | *5* | *5.0143* | *0.0009213 **** |
| Residuals | 0.00105559 | 47 |  |  | Residuals | 0.00105559 | 47 |  |  |
| **Max4.KT.3d20_2d.res.disp** |  |  |  |  | **Max4.KT.3d20_2d.res.disp** |  |  |  |  |
| Watershed | 0.00046992 | 5 | 1.7418 | 0.14360 | Watershed | 0.00028943 | 5 | 1.0728 | 0.38752 |
| LD1 | *0.00036415* | *1* | *6.7487* | *0.01249 ** | simpson | 0.00001474 | 1 | 0.2732 | 0.60366 |
| Watershed:LD1 | 0.00047955 | 5 | 1.7775 | 0.13586 | Watershed:simpson | *0.00082896* | *5* | *3.0726* | *0.01762 ** |
| Residuals | 0.00253603 | 47 |  |  | Residuals | 0.00253603 | 47 |  |  |
| **Mand.openingLR.res.disp** |  |  |  |  | **Mand.openingLR.res.disp** |  |  |  |  |
| Watershed | *0.00031801* | *5* | *5.1648* | *0.0007405 **** | Watershed | *0.00028135* | *5* | *4.5694* | *0.001774 *** |
| LD1 | *0.00007506* | *1* | *6.0951* | *0.0172473 ** | simpson | 0.00000001 | 1 | 0.0007 | 0.979521 |
| Watershed:LD1 | 0.00007264 | 5 | 1.1798 | 0.3333221 | Watershed:simpson | 0.00014769 | 5 | 2.3987 | 0.051133 . |
| Residuals | 0.00057878 | 47 |  |  | Residuals | 0.00057878 | 47 |  |  |
| **Mand.closingLR.res.disp** |  |  |  |  | **Mand.closingLR.res.disp** |  |  |  |  |
| Watershed | *0.00033857* | *5* | *5.2716* | *0.0006348 **** | Watershed | *0.00031019* | *5* | *4.8298* | *0.001207 *** |
| LD1 | *0.00014985* | *1* | *11.6663* | *0.0013213 *** | simpson | 0.00000038 | 1 | 0.0293 | 0.864851 |
| Watershed:LD1 | 0.00009747 | 5 | 1.5176 | 0.2024704 | Watershed:simpson | *0.00024695* | *5* | *3.8450* | *0.005301 *** |
| Residuals | 0.00060371 | 47 |  |  | Residuals | 0.00060371 | 47 |  |  |

| **Table S15.** PERMANOVAs including disparities between biomechanical estimate residuals from 3D and 2D landmark data as dependent variables. All PERMANOVAs include watershed as a factor and diet type (left) or Gini-Simpson diversity (right) as covariates. Model names indicate lever system and rotational axis orientations (Op4º/Max4º; 3d*X*/*X*_2d indicates disparity between model from 3D landmarks with 3D kinematics and model from 2D landmarks with planar kinematics). Effects use type II sums of squares. Significance codes: (.) 0.1, (*) 0.05, (**) 0.01, (***) 0.001. | | | | | | | | | | | | | | | |  |
| --- | --- | --- | --- | --- | --- | --- | --- | --- | --- | --- | --- | --- | --- | --- | --- | --- |
| Mech.disparity | | Df | SS | MS | Rsq | F | Z | Pr(>F) |  | Df | SS | MS | Rsq | F | Z | Pr(>F) |
| Watershed | | *5* | *0.0015178* | *0.00030356* | *0.19852* | *3.0400* | *2.4132* | *0.005 *** | Watershed | *5* | *0.0012290* | *2.4579e-04* | *0.16074* | *2.4615* | *2.00032* | *0.014 ** |
| LD1 | | *1* | *0.0007785* | *0.00077846* | *0.10182* | *7.7960* | *2.1820* | *0.004 *** | simpson | 1 | 0.0000179 | 1.7860e-05 | 0.00234 | 0.1789 | -0.94547 | 0.827 |
| Watershed:LD1 | | 5 | 0.0008019 | 0.00016038 | 0.10489 | 1.6062 | 1.0438 | 0.144 | Watershed:simpson | *5* | *0.0015625* | *3.1250e-04* | *0.20437* | *3.1296* | *2.30639* | *0.007 *** |
| Residuals | | 47 | 0.0046931 | 0.00009985 | 0.61384 |  |  |  | Residuals | 47 | 0.0046931 | 9.9854e-05 | 0.61384 |  |  |  |
| Total | | 58 | 0.0076456 |  |  |  |  |  | Total | 58 | 0.0076456 |  |  |  |  |  |
| Mech.disparity.15º/10º | | | | | | | | | | | | | | | |  |
| Watershed | | *5* | *0.0010743* | *0.00021485* | *0.22416* | *3.7031* | *2.9243* | *0.002 *** | Watershed | *5* | *0.0009179* | *1.8358e-04* | *0.19153* | *3.1641* | *2.57580* | *0.003 *** |
| LD1 | | *1* | *0.0005228* | *0.00052278* | *0.10908* | *9.0104* | *2.6153* | *0.001 *** | simpson | 1 | 0.0000280 | 2.8027e-05 | 0.00585 | 0.4831 | -0.25655 | 0.627 |
| Watershed:LD1 | | 5 | 0.0005351 | 0.00010702 | 0.11165 | 1.8445 | 1.4193 | 0.072 . | Watershed:simpson | *5* | *0.0010298* | *2.0597e-04* | *0.21488* | *3.5499* | *2.72343* | *0.002 *** |
| Residuals | | 47 | 0.0027269 | 0.00005802 | 0.56900 |  |  |  | Residuals | 47 | 0.0027269 | 5.8019e-05 | 0.56900 |  |  |  |
| Total | | 58 | 0.0047925 |  |  |  |  |  | Total | 58 | 0.0047925 |  |  |  |  |  |
| Mech.disparity.30º/20º | | | | | | | | | | | | | | | |  |
| Watershed | | *5* | *0.0017207* | *0.00034413* | *0.19111* | *3.0326* | *2.7184* | *0.002 *** | Watershed | *5* | *0.0015719* | *0.00031437* | *0.17458* | *2.7704* | *2.51081* | *0.004 *** |
| LD1 | | *1* | *0.0010202* | *0.00102023* | *0.11331* | *8.9907* | *2.5781* | *0.001 *** | simpson | 1 | 0.0000838 | 0.00008377 | 0.00930 | 0.7382 | 0.13671 | 0.488 |
| Watershed:LD1 | | 5 | 0.0010194 | 0.00020387 | 0.11322 | 1.7966 | 1.4209 | 0.068 . | Watershed:simpson | *5* | *0.0019558* | *0.00039117* | *0.21723* | *3.4471* | *2.75419* | *0.003 *** |
| Residuals | | 47 | 0.0053334 | 0.00011348 | 0.59236 |  |  |  | Residuals | 47 | 0.0053334 | 0.00011348 | 0.59236 |  |  |  |
| Total | | 58 | 0.0090036 |  |  |  |  |  | Total | 58 | 0.0090036 |  |  |  |  |  |
| Mech.disparity.3d15º/10º_2d.planar | | | | | | | | | | | | | | | |  |
| Watershed | | *5* | *0.0010699* | *0.00021399* | *0.22794* | *3.8128* | *2.9558* | *0.002 *** | Watershed | *5* | *0.0009108* | *1.8217e-04* | *0.19405* | *3.2459* | *2.57994* | *0.003 *** |
| LD1 | | *1* | *0.0005150* | *0.00051499* | *0.10971* | *9.1761* | *2.6017* | *0.001 *** | simpson | 1 | 0.0000353 | 3.5280e-05 | 0.00752 | 0.6286 | 0.00289 | 0.522 |
| Watershed:LD1 | | 5 | 0.0005419 | 0.00010837 | 0.11544 | 1.9310 | 1.4946 | 0.057 . | Watershed:simpson | *5* | *0.0010216* | *2.0432e-04* | *0.21764* | *3.6405* | *2.71770* | *0.001 *** |
| Residuals | | 47 | 0.0026378 | 0.00005612 | 0.56194 |  |  |  | Residuals | 47 | 0.0026378 | 5.6123e-05 | 0.56194 |  |  |  |
| Total | | 58 | 0.0046940 |  |  |  |  |  | Total | 58 | 0.0046940 |  |  |  |  |  |
| Mech.disparity. 3d30º/20º_2d.planar | | | | | | | | | | | | | | | |  |
| Watershed | | *5* | *0.0016153* | *0.00032306* | *0.19643* | *3.0674* | *2.7161* | *0.004 *** | Watershed | *5* | *0.0014675* | *0.00029350* | *0.17845* | *2.7867* | *2.48902* | *0.006 *** |
| LD1 | | *1* | *0.0007529* | *0.00075294* | *0.09156* | *7.1490* | *2.3388* | *0.003 *** | simpson | 1 | 0.0001300 | 0.00012998 | 0.01581 | 1.2342 | 0.63303 | 0.283 |
| Watershed:LD1 | | *5* | *0.0010256* | *0.00020512* | *0.12472* | *1.9476* | *1.5648* | *0.047 ** | Watershed:simpson | *5* | *0.0016486* | *0.00032971* | *0.20047* | *3.1306* | *2.50131* | *0.005 *** |
| Residuals | | 47 | 0.0049501 | 0.00010532 | 0.60194 |  |  |  | Residuals | 47 | 0.0049501 | 0.00010532 | 0.60194 |  |  |  |
| Total | | 58 | 0.0082235 |  |  |  |  |  | Total | 58 | 0.0082235 |  |  |  |  |  |

| **Table S16.** Pairwise comparisons of vector angles and magnitudes between multivariate biomechanical estimates calculated from 2D and 3D landmark and kinematic variables from the same watershed. Diet type and Simpson diversity were used as covariates for vectors. A comparison between 2D and 3D data for all watersheds combined, not grouped by watershed is shown in the bottom row. | | | | | | | | |
| --- | --- | --- | --- | --- | --- | --- | --- | --- |
|  | Watershed*Dimensions*LD | | | | Watershed*Dimensions*Simpson | | | |
| Watershed | Angle (degrees) | p | Proc. distance | p | Angle (degrees) | p | Proc. distance | p |
| Beaver | 5.511131 | 0.9850 | 0.002651395 | 0.9264 | 5.511131 | 0.9821 | 0.06200487 | 0.9332 |
| Boot | 2.335967 | 0.9507 | 0.01991541 | 0.8744 | 2.335967 | 0.9509 | 0.5970575 | 0.8753 |
| Misty | 0.5438605 | 0.9992 | 0.001301694 | 0.9862 | 0.5438605 | 0.9996 | 0.5356883 | 0.9852 |
| Pye | 0.3535929 | 0.9995 | 0.00876852 | 0.9186 | 0.3535929 | 0.9993 | 0.4480557 | 0.9209 |
| Roberts | 1.332877 | 0.9597 | 0.02346502 | 0.8627 | 1.332877 | 0.9610 | 0.5618780 | 0.8632 |
| Village Bay | 0.9605422 | 0.9991 | 0.007911051 | 0.8856 | 0.9605422 | 0.9994 | 0.6409828 | 0.8860 |
|  | Dimensions*LD | | | | Dimensions*Simpson | | | |
| Total 2D-3D comparison | 19.45675 | 0.8146 | 0.003091556 | 0.9023 | 0.5157855 | 0.9986 | 0.0007018907 | 0.9502 |

| **Table S17.** Pairwise comparisons of vector angles between multivariate biomechanical estimates calculated from 2D (rows) and 3D (columns) landmark and kinematic variables from the same watershed. Column and row names indicate landmark dimensionality and rotational axis orientations (Op4º/Max4º) used in kinematic models. Row names also indicate watershed. Diet type was used as a covariate for vectors. | | | | | | |
| --- | --- | --- | --- | --- | --- | --- |
| Watershed*Kin. Model Version*LD | 3D-0 angle (degrees) | p | 3D-15/10 angle (degrees) | p | 3D-30/20 angle (degrees) | p |
| Beaver.2D-0 | 5.51113091 | 0.9818 | 3.597437318 | 0.9953 | 4.946577293 | 0.9904 |
| Boot.2D-0 | 2.335966715 | 0.9488 | 1.732997258 | 0.9733 | 2.049512563 | 0.9621 |
| Misty.2D-0 | 0.543860484 | 0.9989 | 0.886773025 | 0.9963 | 1.72742473 | 0.9759 |
| Pye.2D-0 | 0.353592864 | 0.9993 | 1.054158275 | 0.9832 | 1.860756158 | 0.9331 |
| Roberts.2D-0 | 1.332877397 | 0.9614 | 1.748795026 | 0.9276 | 3.623885744 | 0.6916 |
| VillageBay.2D-0 | 0.960542248 | 0.9995 | 3.268925101 | 0.9815 | 6.138271535 | 0.9237 |
| Beaver.2D-15/10 | 5.736759648 | 0.9817 | 3.841737391 | 0.9923 | 5.157219762 | 0.9859 |
| Boot.2D-15/10 | 2.260571873 | 0.9516 | 1.627592638 | 0.9776 | 2.003703741 | 0.9634 |
| Misty.2D-15/10 | 0.465456411 | 0.9991 | 1.042335478 | 0.9940 | 1.898531162 | 0.9697 |
| Pye.2D-15/10 | 0.25841414 | 0.9998 | 1.211960619 | 0.9742 | 2.022371773 | 0.9175 |
| Roberts.2D-15/10 | 1.328987611 | 0.9614 | 1.755028316 | 0.9221 | 3.630815747 | 0.6842 |
| VillageBay.2D-15/10 | 0.766937288 | 0.9998 | 3.798162516 | 0.9747 | 6.681756092 | 0.9133 |
| Beaver.2D-30/20 | 6.362667125 | 0.9765 | 4.527180127 | 0.9902 | 5.754732704 | 0.9859 |
| Boot.2D-30/20 | 2.120191051 | 0.9601 | 1.421065505 | 0.9843 | 1.945348359 | 0.9668 |
| Misty.2D-30/20 | 0.558652192 | 0.9993 | 1.483552706 | 0.9854 | 2.362089916 | 0.9509 |
| Pye.2D-30/20 | 0.427334399 | 0.9983 | 1.655083896 | 0.9457 | 2.471828051 | 0.8669 |
| Roberts.2D-30/20 | 1.316971992 | 0.9626 | 1.794358324 | 0.9230 | 3.67302817 | 0.6838 |
| VillageBay.2D-30/20 | 1.663370696 | 0.9975 | 5.275535365 | 0.9437 | 8.181673533 | 0.8726 |

| **Table S18.** Pairwise comparisons of vector magnitudes between multivariate biomechanical estimates calculated from 2D (rows) and 3D (columns) landmark and kinematic variables from the same watershed. Column and row names indicate landmark dimensionality and rotational axis orientations (Op4º/Max4º) used in kinematic models. Row names also indicate watershed. Diet type was used as a covariate for vectors. | | | | | | |
| --- | --- | --- | --- | --- | --- | --- |
| Watershed*Kin. Model Version*LD | 3D-0 distance | P | 3D-15/10 distance | p | 3D-30/20 distance | p |
| Beaver.2D-0 | 0.002651395 | 0.9336 | 0.002214449 | 0.9418 | 0.002971901 | 0.9241 |
| Boot.2D-0 | 0.019915406 | 0.8681 | 0.017139846 | 0.8880 | 0.019294995 | 0.8814 |
| Misty.2D-0 | 0.001301694 | 0.9856 | 0.000256306 | 0.9974 | 0.000997915 | 0.9895 |
| Pye.2D-0 | 0.00876852 | 0.9227 | 0.006299761 | 0.9446 | 0.003214765 | 0.9694 |
| Roberts.2D-0 | 0.023465016 | 0.8610 | 0.014566001 | 0.9145 | 0.011855543 | 0.9307 |
| VillageBay.2D-0 | 0.007911051 | 0.8962 | 0.005832311 | 0.9207 | 0.004188784 | 0.9360 |
| Beaver.2D-15/10 | 0.002368232 | 0.9395 | 0.001931286 | 0.9522 | 0.002688738 | 0.9355 |
| Boot.2D-15/10 | 0.019805771 | 0.8729 | 0.017030211 | 0.8905 | 0.01918536 | 0.8759 |
| Misty.2D-15/10 | 0.001105842 | 0.9876 | 6.05E-05 | 0.9997 | 0.001193768 | 0.9854 |
| Pye.2D-15/10 | 0.00831345 | 0.9215 | 0.00584469 | 0.9452 | 0.002759694 | 0.9745 |
| Roberts.2D-15/10 | 0.023414226 | 0.8583 | 0.014515211 | 0.9164 | 0.011804752 | 0.9284 |
| VillageBay.2D-15/10 | 0.007596424 | 0.8933 | 0.005517684 | 0.9283 | 0.003874156 | 0.9442 |
| Beaver.2D-30/20 | 0.001602942 | 0.9607 | 0.001165995 | 0.9717 | 0.001923448 | 0.9545 |
| Boot.2D-30/20 | 0.019545852 | 0.8713 | 0.016770292 | 0.8886 | 0.018925441 | 0.8790 |
| Misty.2D-30/20 | 0.000590107 | 0.9939 | 0.000455281 | 0.9952 | 0.001709503 | 0.9825 |
| Pye.2D-30/20 | 0.007031084 | 0.9348 | 0.004562325 | 0.9588 | 0.001477329 | 0.9853 |
| Roberts.2D-30/20 | 0.023178467 | 0.8584 | 0.014279452 | 0.9165 | 0.011568994 | 0.9310 |
| VillageBay.2D-30/20 | 0.006684333 | 0.9100 | 0.004605593 | 0.9344 | 0.002962065 | 0.9580 |

| **Table S19.** Pairwise comparisons of vector angles between multivariate biomechanical estimates calculated from 2D (rows) and 3D (columns) landmark and kinematic variables from the same watershed. Column and row names indicate landmark dimensionality and rotational axis orientations (Op4º/Max4º) used in kinematic models. Row names also indicate watershed. Diet diversity was used as a covariate for vectors. | | | | | | |
| --- | --- | --- | --- | --- | --- | --- |
| Watershed*Kin. Model Version*simpson_ | 3D-0 angle (degrees) | p | 3D-15/10 angle (degrees) | p | 3D-30/20 angle (degrees) | p |
| Beaver.2D-0 | 5.51113091 | 0.9834 | 3.597437318 | 0.9943 | 4.946577293 | 0.9897 |
| Boot.2D-0 | 2.335966715 | 0.9464 | 1.732997258 | 0.9724 | 2.049512563 | 0.9617 |
| Misty.2D-0 | 0.543860484 | 0.9982 | 0.886773025 | 0.9962 | 1.72742473 | 0.9730 |
| Pye.2D-0 | 0.353592864 | 0.9992 | 1.054158275 | 0.9822 | 1.860756158 | 0.9262 |
| Roberts.2D-0 | 1.332877397 | 0.9622 | 1.748795026 | 0.9269 | 3.623885744 | 0.6851 |
| VillageBay.2D-0 | 0.960542248 | 0.9998 | 3.268925101 | 0.9809 | 6.138271535 | 0.9147 |
| Beaver.2D-15/10 | 5.736759648 | 0.9815 | 3.841737391 | 0.9932 | 5.157219762 | 0.9854 |
| Boot.2D-15/10 | 2.260571873 | 0.9510 | 1.627592638 | 0.9781 | 2.003703741 | 0.9622 |
| Misty.2D-15/10 | 0.465456411 | 0.9994 | 1.042335478 | 0.9933 | 1.898531162 | 0.9656 |
| Pye.2D-15/10 | 0.25841414 | 0.9993 | 1.211960619 | 0.9709 | 2.022371773 | 0.9098 |
| Roberts.2D-15/10 | 1.328987611 | 0.9620 | 1.755028316 | 0.9211 | 3.630815747 | 0.6804 |
| VillageBay.2D-15/10 | 0.766937288 | 0.9996 | 3.798162516 | 0.9734 | 6.681756092 | 0.9083 |
| Beaver.2D-30/20 | 6.362667125 | 0.9762 | 4.527180127 | 0.9884 | 5.754732704 | 0.9814 |
| Boot.2D-30/20 | 2.120191051 | 0.9593 | 1.421065505 | 0.9850 | 1.945348359 | 0.9677 |
| Misty.2D-30/20 | 0.558652192 | 0.9984 | 1.483552706 | 0.9807 | 2.362089916 | 0.9457 |
| Pye.2D-30/20 | 0.427334399 | 0.9987 | 1.655083896 | 0.9425 | 2.471828051 | 0.8577 |
| Roberts.2D-30/20 | 1.316971992 | 0.9644 | 1.794358324 | 0.9203 | 3.67302817 | 0.6732 |
| VillageBay.2D-30/20 | 1.663370696 | 0.9973 | 5.275535365 | 0.9402 | 8.181673533 | 0.8536 |

| **Table S20.** Pairwise comparisons of slope vector lengths between multivariate biomechanical estimates calculated from 2D (rows) and 3D (columns) landmark and kinematic variables from the same watershed. Column and row names indicate landmark dimensionality and rotational axis orientations (Op4º/Max4º) used in kinematic models. Row names also indicate watershed. Diet diversity was used as a covariate for vectors. | | | | | | |
| --- | --- | --- | --- | --- | --- | --- |
| Watershed*Kin. Model Version*simpson | 3D-0 distance | p | 3D-15/10 distance | p | 3D-30/20 distance | p |
| Beaver.2D-0 | 0.062004872 | 0.9315 | 0.051786553 | 0.9435 | 0.069500149 | 0.9206 |
| Boot.2D-0 | 0.59705753 | 0.8674 | 0.513847128 | 0.8893 | 0.578457808 | 0.8804 |
| Misty.2D-0 | 0.535688293 | 0.9852 | 0.105478165 | 0.9972 | 0.410673586 | 0.9873 |
| Pye.2D-0 | 0.448055734 | 0.9251 | 0.321906549 | 0.9448 | 0.164268764 | 0.9708 |
| Roberts.2D-0 | 0.561877969 | 0.8586 | 0.348787957 | 0.9152 | 0.283885089 | 0.9297 |
| VillageBay.2D-0 | 0.640982763 | 0.8917 | 0.47255552 | 0.9223 | 0.33939082 | 0.9393 |
| Beaver.2D-15/10 | 0.055382888 | 0.9392 | 0.045164569 | 0.9489 | 0.062878165 | 0.9292 |
| Boot.2D-15/10 | 0.593770692 | 0.8742 | 0.51056029 | 0.8894 | 0.57517097 | 0.8754 |
| Misty.2D-15/10 | 0.455088896 | 0.9879 | 0.024878768 | 0.9994 | 0.491272983 | 0.9852 |
| Pye.2D-15/10 | 0.424802427 | 0.9196 | 0.298653242 | 0.9430 | 0.141015456 | 0.9742 |
| Roberts.2D-15/10 | 0.560661775 | 0.8578 | 0.347571762 | 0.9147 | 0.282668895 | 0.9262 |
| VillageBay.2D-15/10 | 0.615490493 | 0.8915 | 0.447063251 | 0.9262 | 0.31389855 | 0.9454 |
| Beaver.2D-30/20 | 0.037485996 | 0.9602 | 0.027267677 | 0.9682 | 0.044981273 | 0.9531 |
| Boot.2D-30/20 | 0.585978411 | 0.8717 | 0.502768009 | 0.8870 | 0.567378689 | 0.8801 |
| Misty.2D-30/20 | 0.242847648 | 0.9935 | 0.18736248 | 0.9959 | 0.703514231 | 0.9796 |
| Pye.2D-30/20 | 0.359275833 | 0.9330 | 0.233126648 | 0.9609 | 0.075488862 | 0.9867 |
| Roberts.2D-30/20 | 0.555016457 | 0.8621 | 0.341926444 | 0.9150 | 0.277023577 | 0.9301 |
| VillageBay.2D-30/20 | 0.541589478 | 0.9070 | 0.373162236 | 0.9391 | 0.239997535 | 0.9604 |

| **Table S21.** Pairwise comparisons of vector angles between multivariate biomechanical estimates calculated from 2D (rows) and 3D (columns) landmark and kinematic variables across all individuals. Column and row names indicate landmark dimensionality and rotational axis orientations (Op4º/Max4º) used in kinematic models. Diet type was used as a covariate for vectors. | | | | | | |
| --- | --- | --- | --- | --- | --- | --- |
| Kin. Model Version *LD | 3D-0 angle (degrees) | p | 3D-15/10 angle (degrees) | p | 3D-30/20 angle (degrees) | p |
| 2D-0 | 19.45675134 | 0.8202 | 20.45479675 | 0.8103 | 24.93788173 | 0.7605 |
| 2D-15/10 | 18.920433 | 0.8301 | 20.3304261 | 0.8122 | 25.1928552 | 0.7571 |
| 2D-30/20 | 17.88525916 | 0.8385 | 20.33096745 | 0.8119 | 26.04803092 | 0.7476 |

| **Table S22.** Pairwise comparisons of slope vector lengths between multivariate biomechanical estimates calculated from 2D (rows) and 3D (columns) landmark and kinematic variables across all individuals. Column and row names indicate landmark dimensionality and rotational axis orientations (Op4º/Max4º) used in kinematic models. Diet type was used as a covariate for vectors. | | | | | | |
| --- | --- | --- | --- | --- | --- | --- |
| Kin. Model Version *LD_dist | 3D-0 distance | p | 3D-15/10 distance | p | 3D-30/20 distance | p |
| 2D-0 | 0.003091556 | 0.9001 | 2.94E-05 | 0.9994 | 0.002085356 | 0.9345 |
| 2D-15/10 | 0.002612183 | 0.9161 | 0.000449952 | 0.9859 | 0.002564729 | 0.9151 |
| 2D-30/20 | 0.001251214 | 0.9592 | 0.001810921 | 0.9385 | 0.003925698 | 0.8738 |

| **Table S23.** Pairwise comparisons of vector angles between multivariate biomechanical estimates calculated from 2D (rows) and 3D (columns) landmark and kinematic variables across all individuals. Column and row names indicate landmark dimensionality and rotational axis orientations (Op4º/Max4º) used in kinematic models. Diet diversity was used as a covariate for vectors. | | | | | | |
| --- | --- | --- | --- | --- | --- | --- |
| Kin. Model Version *simpson | 3D-0 angle (degrees) | p | 3D-15/10 angle (degrees) | p | 3D-30/20 angle (degrees) | p |
| 2D-0 | 0.515785467 | 0.9977 | 0.298795789 | 0.9994 | 0.46138021 | 0.9981 |
| 2D-15/10 | 0.529648554 | 0.9971 | 0.256186573 | 0.9997 | 0.45229454 | 0.9984 |
| 2D-30/20 | 0.603637984 | 0.9980 | 0.251918922 | 1.0000 | 0.486764336 | 0.9984 |

| **Table S24.** Pairwise comparisons of slope vector lengths between multivariate biomechanical estimates calculated from 2D (rows) and 3D (columns) landmark and kinematic variables across all individuals. Column and row names indicate landmark dimensionality and rotational axis orientations (Op4º/Max4º) used in kinematic models. Diet diversity was used as a covariate for vectors. | | | | | | |
| --- | --- | --- | --- | --- | --- | --- |
| Kin. Model Version *simpson_dist | 3D-0 distance | p | 3D-15/10 distance | p | 3D-30/20 distance | p |
| 2D-0 | 0.066141361 | 0.9512 | 0.059692148 | 0.9551 | 0.066029593 | 0.9513 |
| 2D-15/10 | 0.064971337 | 0.9554 | 0.058522123 | 0.9591 | 0.064859568 | 0.9515 |
| 2D-30/20 | 0.062340587 | 0.9525 | 0.055891374 | 0.9527 | 0.062228819 | 0.9516 |

| Table S25. Parallel / non-parallel effect size ratios of diet type, habitat, and diversity on KT or LR in 2D and 3D ANCOVAs. For Op4 and Max4 linkages numbers after period indicate rotational axis angles as specified in Methods. Effect sizes used for ratios are partial Eta^2^ values (SS_effect_/[SS_effect_+SS_error_]). | | | | | | |
| --- | --- | --- | --- | --- | --- | --- |
| Biomechanical Model | 2D Diet LD | 3D Diet LD | 2D Habitat | 3D Habitat | 2D Simpson Div. | 3D Simpson Div. |
| Op4 | 0.27 | 0.31 | 0.36 | 0.42 | 0.10 | 0.09 |
| Op4.15 | 0.28 | 0.26 | 0.37 | 0.36 | 0.10 | 0.08 |
| Op4.30 | 0.30 | 0.22 | 0.40 | 0.31 | 0.09 | 0.08 |
| Mand Opening | 0.08 | 0.10 | 0.11 | 0.13 | 0.31 | 0.29 |
| Mand Closing | 0.01 | 0.02 | 0.01 | 0.02 | 0.34 | 0.33 |
| Max4 | 0.04 | 0.07 | 0.05 | 0.09 | 0.33 | 0.30 |
| Max4.10 | 0.04 | 0.06 | 0.05 | 0.08 | 0.33 | 0.31 |
| Max4.20 | 0.04 | 0.07 | 0.05 | 0.09 | 0.33 | 0.30 |
